# Current Progress and Future Directions in the Double Burden of Malnutrition among Women in South and Southeast Asian Countries

**DOI:** 10.1093/cdn/nzz026

**Published:** 2019-05-16

**Authors:** Tuhin Biswas, Nick Townsend, R J Soares Magalhaes, Md Saimul Islam, Md Mehedi Hasan, Abdullah Mamun

**Affiliations:** 1 Institute for Social Science Research, The University of Queensland, Brisbane, Australia; 2 ARC Centre of Excellence for Children and Families over the Life Course, The University of Queensland, Indooroopilly, Australia; 3 UQ Spatial Epidemiology Laboratory, School of Veterinary Science, The University of Queensland, Gatton, Australia; 4 Children's Health and Environment Program, Child Health Research Centre, The University of Queensland, Brisbane, Australia; 5 Public Health Epidemiology, Department for Health, University of Bath, Bath, United Kingdom; 6 Department of Statistics, University of Rajshahi, Rajshahi, Bangladesh

**Keywords:** malnutrition, SDG2, women, South and Southeast Asia

## Abstract

**Background:**

In order to combat the double burden of malnutrition the UN General Assembly has established under its Sustainable Development Goal-2 (SDG2) a set of nutritional targets that member countries need to achieve by 2030, with the goal of eradicating all forms of malnutrition worldwide.

**Objectives:**

In order to understand progress towards this goal, we reviewed recent trends and forecast future trends to examine the likelihood of South and Southeast Asian countries achieving the SDG2 target by 2030. We also considered how inequalities based on wealth, education, and urban/rural dwelling influence the current and future prevalence of underweight, overweight, and obesity.

**Methods:**

We used population-representative cross-sectional data from the Demographic and Health Survey, conducted between 1996 and 2016, for 8 South and Southeast Asian countries. We used a Bayesian linear regression model to estimate trends and to forecast the prevalence of underweight, overweight, and obesity by 2030.

**Results:**

The overall pooled prevalence of underweight, overweight, and obesity in the South and Southeast Asian region was 22.9%, 21.3%, and 8.6%, respectively. Regional average annual rate of reduction and average annual rate of increase for the period 1996 to 2016 were 1.3% and 8.4% for underweight and overweight/obesity respectively. We estimate that if current trends continue as projected, the proportion of underweight and overweight/obesity will be 6.6% (95% CI: 3.9%, 11.1%) and 76.6% (95% CI: 64.3%, 85.7%) in 2030, respectively. Specific projections based on the wealth index suggested that by 2030 the prevalence of underweight would be highest among the poorest sector of society, and overweight and obesity highest among the richest sector.

**Conclusions:**

We found that despite progress in reducing underweight, nearly two-thirds of the South and Southeast Asian population will be overweight or obese by 2030. Our findings suggest that countries in this region will not achieve the 2030 SDG2 target.

## Introduction

The double burden of malnutrition (DBM) has been identified as one of the most important contemporary global health challenges (1). The paradox is characterized by the coexistence of undernutrition along with overweight and obesity within individuals, households, and populations, across the life course (2, 3). The DBM is of particular concern among women of reproductive age in South and Southeast Asia because overweight is increasing in this group despite maternal undernutrition still being prevalent (4). A recent systematic review and meta-analysis covering the region reported that the current prevalence of overweight actually exceeds that of underweight. This is concerning, as it is well known that both underweight and overweight have multifaceted consequences for survival, incidence of chronic diseases, healthy development, and the economic productivity of individuals, societies, and health care systems (5, 6). For example, undernutrition in women is associated with adverse pregnency outcomes, including maternal mortality, delivery complications, preterm birth, and intrauterine growth retardation (7).

Likewise, maternal overweight and obesity leads to several adverse maternal and fetal complications during pregnancy, delivery, and postpartum (8). With the nutrition status of women seen to be closely linked to that of their children, improving the health of women and children begins with ensuring the health and nutritional status of women throughout the life course (9).

In recent decades underweight in women has declined considerably in most of the low- and middle-income countries (LMICs), with many of these countries experiencing a more rapidly increasing prevalence of overweight and obesity in women than previously thought (10). At the same time, some determinants of both underweight and overweight in LMICs have been identified. For example overweight and obesity in women is more common in women of higher socioeconomic status and higher education (11–14); whereas low socioeconomic status and low education continues to be a strong determinant of underweight (12, 15). However, in South and Southeast Asia there is no clear evidence on which demographic groups are experiencing a great burden from both underweight and overweight/obesity in women. In particular, understanding the relative rates of change in the prevalence of underweight and overweight/obesity, both overall and within subgroups, in South and Southeast Asia has important policy implications, as does identifying the determinants of these changes.

In recognition of this, the UN General Assembly defined a set of nutritional targets that member countries need to achieve by 2030, as the second of 17 Sustainable Development Goals (SDG2) (16). These include the target to eradicate all forms of malnutrition worldwide. All country leaders within this region have committed to achieving SDG2, hence combating the DBM (17). Continued surveillance and monitoring of both underweight and overweight are crucial to follow progress in achieving this. In addition, projections of future trends would also prove informative for public health policy-makers as they seek to achieve time-bound goals in malnutrition. Despite this, no previous study on South and Southeast Asia has described past trends and future projections in the DBM within individual countries and the region as a whole.

In this study, we aimed to investigate the trends and determinants of the DBM, as well as modeling forecasting trends of the DBM to 2030 for South and Southeast Asian countries. In doing so, we also estimated the average annual rate of reduction (AARR) and the average annual rate of increase (AARI) for underweight and overweight/obesity within countries, by selected subgroup characteristics.

## Methods

### Data sources and procedures

We used population-representative cross-sectional data from the Demographic and Health Survey (DHS), conducted between 1996 and 2016, for 8 South and Southeast Asian countries: Bangladesh, India, Nepal, Pakistan, Myanmar, Timor-Leste, Maldives, and Cambodia. These surveys were conducted at 5-y intervals across LMICs. They use standardized data collection and sampling procedures in all countries. This includes a stratified 2-stage random sampling approach, consisting of a selection of census enumeration areas based on a probability, followed by a random selection of household from a complete listing of households within the selected enumeration areas. Health- and welfare-related data were collected through interviewing women of reproductive age (15–49 y) within households. Following standard protocols, written informed consent was obtained from all participants before interviews were conducted. Ethical approval was given by the ICF International (Calverton, MD) institutional review board and by individual review boards within each participating country. The DHS dataset can be obtained from the DHS program website according to a data-sharing policy (18). Detailed descriptions of DHS sampling procedures, validation of questionnaires, and data collection methods are published elsewhere (18). In this study, we have used data for our select population of women of reproductive age, including anthropometric measurements (weight in kg and height in cm), along with other health and welfare data. These corresponded to >1 million women from all 8 countries over the period examined.

### Measurements

In all DHS surveys, trained personnel take height and weight measurements following a standardized procedure. Weight was measured with solar-powered scales accurate to 0.1 kg and height was measured with standardized measuring boards accurate to 0.1 cm. BMI was calculated as weight (kg)/height (m^2^). Asian-specific BMI cutoffs were used to define underweight (<18.5), overweight (23.0–<27.5), and obese (≥27.5) as recommended by the WHO (19) and used in a number of studies (11, 12) within this population (20). Because the prevalence of obesity was low in some countries, the categories of overweight and obesity were combined together in the AARR, AARI, multinominal logistic regression analysis, and projection. Only respondents for whom the required information to calculate BMI was available were included in the analyses.

The participants’ place of residence was designated as rural or urban according to country-specific definitions. The wealth index is a composite score calculated by DHS staff from principal components analysis on routinely collected data on a household's ownership of selected assets, including televisions, bicycles, cars, materials used for housing construction, types of water access, sanitation facilities, and types of fuel used. This continuous scale of relative wealth was then categorized into 5 levels: poorest (quintile 1), poorer (quintile 2), middle (quintile 3), richer (quintile 4), and richest (quintile 5), according to the quintiles calculated from the asset variables. Levels of education were categorized as no education (indicating 0 grade), primary education (indicating completed grade 1–5), secondary education (indicting completed grade 6–10), and higher education (indicating completed higher than grade 10).

### Statistical analysis

We have used descriptive statistics to summarize the continuous variables and frequency distribution to summarize the categoric variables. For the purpose of the analysis, the average annual rate of change, namely the AARI and the AARR for overweight/obesity and underweight prevalence, respectively, among women between 2 consecutive DHS surveys was calculated from the following formula:

\begin{equation*}
{Y_{t + n}} = {Y_{\rm{t}}} \times {\left( {1 \pm b\% } \right)^n}
\end{equation*}

in which *Y_t_* = the prevalence of underweight and overweight/obesity of any given year *t*; *b* = annual rate of change; *n* = number of years between 2 surveys, and *Y_t_*_+_*_n_* = the prevalence of underweight and overweight/obesity in the (*t* + *n*)th year. This information is provided in a UNICEF technical note (21).

Multinominal logistic regression was used to estimate the ORs of being underweight and overweight/obese, with individuals of normal weight used as the reference group. For region-specific analysis, data were pooled to combine all countries and all years of survey data. The adjusted ORs were then estimated, considering all potential determinants in the final model. Country-specific analysis was done for those countries for which we had multiple time point data. All analyses were adjusted for cluster and sample weight.

We applied Bayesian linear regression models with noninformative priors to estimate the trends in indicators with time and the posterior predictive distribution of the indicators (22). Some countries had no recent data and the projections have been made forward to the year 2016. We have estimated the projected prevalence over time at 2030 by place of residence, wealth index, and education. Bayesian linear regression specifies a sampling distribution of the data with specification of a prior distribution of the regression coefficients. All the proportions were logit transformed before the analysis, and all calculations were done in the logit-transformed variables and then transformed back to probabilities to ensure all predicted probabilities lay between 0 and 1. To analyze the Bayesian model through the use of a Markov chain Monte Carlo approach, we used the R programming language and JAGS, a BUGS language. A Markov chain Monte Carlo algorithm was used to obtain 1000 samples from the posterior distributions of the parameter of interest with 2 chains. For each model, the first 5000 iterations were discarded as burn-in and the number of iterations was increased until the output was diagnosed as converged. Trace plots were generated to verify mixing, and the chains were past the burn-in phase. These posterior predictive distributions were used to obtain projections and credible intervals up to the year 2030. Country-specific projections were only conducted for Bangladesh and Nepal, as these were the only countries for which we had multiple time point data.

## Results

### General characteristics

Data from 1,085,924 women from 8 countries in South and Southeast Asia were analyzed in this study. **Table 1** shows the country-level characteristics of the study participants. The majority of the sample came from India (*n* = 824,072), followed by Nepal (*n* = 94,470), and Bangladesh (*n* = 67,516). The overall mean age of the study participants was 29.8 y, ranging from a mean of 28.6 y in Nepal (2006) to 30.8 y in Bangladesh (2011). On average, 31.7% of the women in the total sample resided in urban areas, ranging from 14.6% in the Maldives to 46.8% in Pakistan. Bangladesh (pooled data) had the lowest percentage of women in the poorest quintile of the wealth index (18.2%), and the Maldives (22.2%) had the highest. The highest proportion of women in the richest quintile (27.2%) was found in Cambodia, and the lowest was found in Timor (9.4%). The largest percentage of women with no education (54.7%) was found in Nepal, and the highest percentage of women with a higher education (12.4%) was found in Pakistan.

**TABLE 1:** General characteristics of the study population

Country	Year	Total sample, *n*	Mean age ± SD, y	Urban residents, %	Lowest household wealth quintile, %	Highest household wealth quintile, %	No education, %	Higher education, %
Bangladesh	1996	9127	29.2 ± 9.0	15.9	20.6	19.2	53.7	3.2
	2000	10,544	29.7 ± 9.3	29.9	20.5	23.3	43.4	5.3
	2004	1144	30.0 ± 9.4	34.1	17.9	25.4	38.6	6.0
	2007	10,996	30.6 ± 9.3	37.8	16.1	26.6	32.1	7.8
	2011	17,842	30.8 ± 9.2	34.7	17.4	23.5	26.0	8.2
	2014	17,863	31.0 ± 9.2	34.5	18.2	21.6	23.5	9.6
	Pooled	67,516	30.3 ± 9.3	32.2	18.2	23.3	33.8	7.2
India	2005	124,386	29.1 ± 9.4	45.8	11.3	31.2	32.0	10.4
	2016	699,686	29.8 ± 9.7	29.3	19.0	18.8	28.1	11.4
	Pooled	824,072	29.7 ± 9.7	31.8	17.9	20.6	28.7	11.3
Nepal	1996	8429	30.6 ± 9.1	11.3	19.4	21.0	79.9	1.3
	2001	8726	30.8 ± 9.0	13.2	19.6	22.6	71.8	1.3
	2006	10,793	28.6 ± 9.7	27.3	20.4	21.7	52.6	4.5
	2011	12,674	28.7 ± 9.6	29.2	19.3	24.3	38.5	8.4
	2016	53,848	29.4 ± 9.5	31.9	20.1	21.4	52.2	6.7
	Pooled	94,470	29.5 ± 9.5	27.4	19.9	21.9	54.7	5.7
Myanmar	2015	12,885	31.6 ± 9.8	29.4	18.3	20.9	12.4	10.3
Pakistan	2012	13,558	32.6 ± 8.5	46.8	18.3	23.9	56.2	12.4
Timor	2009	13,137	28.6 ± 10.0	24.6	19.4	19.0	29.9	2.3
Maldives	2009	7131	32.5 ± 8.3	14.6	22.2	9.3	27.2	3.0
Cambodia	2005	16,823	29.6 ± 10.2	24.7	19.4	23.0	22.4	0.9
	2010	18,754	29.5 ± 10.1	32.4	17.4	28.6	17.1	3.3
	2014	17,578	30.1 ± 9.8	32.2	17.4	29.7	12.7	5.6
	Pooled	53,155	29.7 ± 10.1	29.9	18.0	27.2	17.3	3.3

### Overall prevalence

**FIGURE 1:**
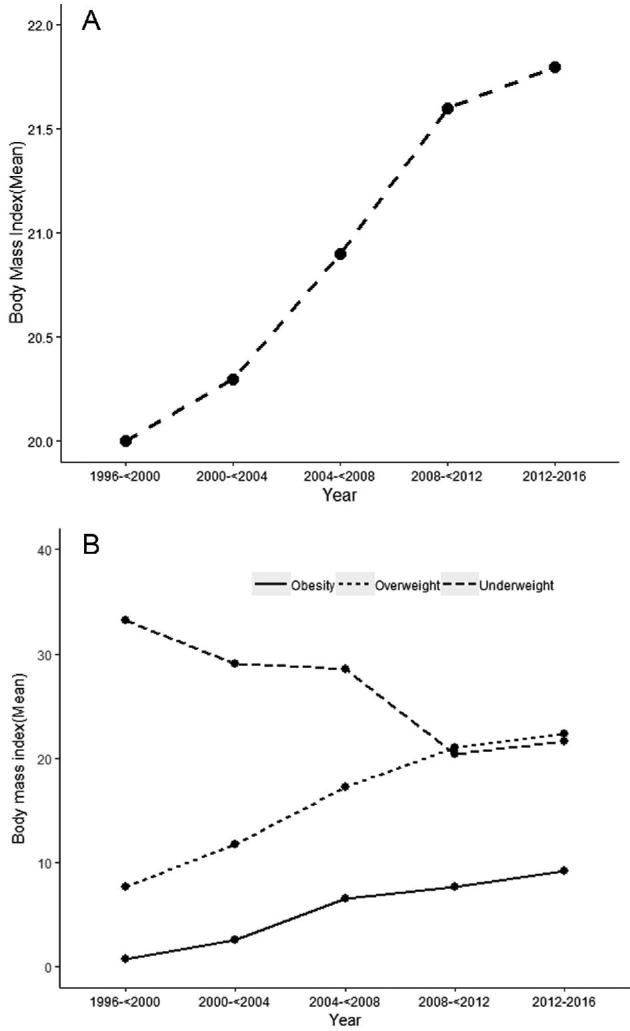
Weight trends in South and Southeast Asia. (A) Trends in distribution of mean BMI in South and Southeast Asia. (B) Trends in prevalence of underweight, overweight, and obesity in South and Southeast Asia.

The mean BMI of the sample population increased from 20.0 prior to 2000 to 21.8 in 2012–2016 (Figure 1A). This corresponds to an increase in the prevalence of obesity from 0.7% to 9.2% and an increase in the prevalence of overweight, including obesity, from 7.7% to 22.3% over the same period of time. Conversely the prevalence of underweight decreased from 33.2% in 1996–1999 to 20.4% in 2008–2011; there was then a small rise to 21.6% in 2012–2016 (Figure 1B). The highest prevalence of underweight was found in Bangladesh in 1996; however, the only other country to provide these data for before 2005 was Nepal. Considering data after 2012 only, India had the highest prevalence of underweight (22.5%) and Cambodia the lowest (15.5%). The highest prevalence of overweight was found in the Maldives (37.8%), the lowest in Bangladesh (5.1%). Considering data after 2012 only, Pakistan had the highest prevalence (29.4%) of overweight, and the lowest was found in India (22.7%). Decreasing trends in underweight and increasing trends in overweight prevalence over this period of time were also found within countries for which numerous data points were available, including Bangladesh, India, Nepal, and Cambodia **(Supplementary Figure 1)**.

### Average annual rate of change

The region-specific estimated AARR and AARI for the prevalence of underweight and overweight/obesity for the period 1996–2014 were 1.3% and 8.4%, respectively. Variation was found in these rates of change between countries. The overall national change in underweight and overweight/obesity for the period 1996–2014 in Bangladesh were an AARR of 6.0% and an AARI of 11.0%, respectively; this compared to an AARR of underweight of 3.0% and an AARI of overweight/obesity of 2.0% in India, an underweight AARR of 2.0% and an overweight/obesity AARI of 7.0% in Nepal, and an underweight AARR and an overweight/obesity AARI of 4.0% and 1.8%, respectively, in Cambodia **(Supplemental Tables 1–8)**. Supplemental Table 1 also shows the overall AARR and AARI of underweight and overweight/obesity by different sociodemographic characteristics (type of residence, wealth index, education, and age group) for Bangladesh, India, Nepal, and Cambodia. In Bangladesh, the AARR for underweight was larger in urban areas, amongst wealthier families, and for those who had a higher education. However, completely the opposite was found for overweight/obesity.

### Determinants of the DBM

Multinomial logistic regression of the total dataset pooled across years and countries involved data from 1085,924 women. In the final models, adjusted for all determinant variables and year of data collection, women who lived in urban areas were 1.29 (95% CI: 1.27, 1.31) times more likely to be overweight or obese and 0.99 (95% CI: 0.98, 1.00) times less likely to be underweight than those from rural areas. Women who lived in the wealthiest households were 4.41 (95% CI: 4.32, 4.51) times more likely to be overweight or obese and 0.51 (95% CI: 0.5, 0.53) times less likely to be underweight compared with women in the poorest households. Having no education was associated with the highest risk of underweight and the lowest risk of overweight and obesity, whereas older women were most likely to be overweight or obese. Those in the youngest age category (15-19 y) were the most likely to be underweight. Although there was no clear trend in the odds of being underweight over time (the highest odds were found in 2004–2007 and the lowest in 2008–2012), there was a constant increase in the odds of being overweight or obese from 1996 to 2016 (**Table 2**). Country-specific information presented in **SupplementalTables9–16.**

**TABLE 2:** Multinominal logistic regression analysis of odds ratio (OR) of being underweight and being overweight compared with normal BMI category (*n* = 1,085,924)^1^

	Underweight				Overweight/obesity			
Variables	Unadjusted	*P* value	Adjusted	*P* value	Unadjusted	*P* value	Adjusted	*P* value
Type of residence								
Urban	0.88 (0.79, 0.81)	0.00	0.99 (0.98, 1)	0.12	2.07 (2.05, 2.09)	0.00	1.29 (1.28, 1.31)	0.00
Rural	Ref.							
Wealth index								
Quintile 5	0.51 (0.5, 0.52)	0.00	0.51 (0.5, 0.53)	0.00	5.17 (5.08, 5.26)	0.00	4.41 (4.32, 4.51)	0.00
Quintile 4	0.65 (0.64, 0.67)	0.00	0.66 (0.65, 0.67)	0.00	3.47 (3.41, 3.53)	0.00	3.13 (3.07, 3.2)	0.00
Quintile 3	0.74 (0.73, 0.75)	0.00	0.75 (0.73, 0.76)	0.00	2.37 (2.33, 2.41)	0.00	2.25 (2.21, 2.29)	0.00
Quintile 2	0.85 (0.84, 0.87)	0.00	0.86 (0.85, 0.87)	0.00	1.61 (1.58, 1.64)	0.00	1.57 (1.54, 1.6)	0.00
Quintile 1	Ref.							
Education								
Higher	0.64 (0.62, 0.65)	0.00	0.77 (0.75, 0.79)	0.00	1.88 (1.85, 1.91)	0.00	1.31 (1.27, 1.33)	0.00
Secondary	0.92 (0.91, 0.93)	0.00	0.84 (0.83, 0.85)	0.00	1.34 (1.33, 1.36)	0.00	1.40 (1.38, 1.42)	0.00
Primary	0.87 (0.86, 0.89)	0.00	0.85 (0.83, 0.86)	0.00	1.26 (1.24, 1.28)	0.00	1.23 (1.21, 1.25)	0.00
No education	Ref							
Age, y								
45–49	0.56 (0.55, 0.57)	0.00	0.49 (0.48, 0.5)	0.00	6.63 (6.49, 6.78)	0.00	8.28 (8.09, 8.48)	0.00
40–44	0.55 (0.54, 0.57)	0.00	0.49 (0.48, 0.5)	0.00	6.27 (6.14, 6.41)	0.00	7.77 (7.59, 7.95)	0.00
35–39	0.53 (0.52, 0.54)	0.00	0.47 (0.46, 0.48)	0.00	5.46 (5.34, 5.57)	0.00	6.65 (6.5, 6.8)	0.00
30–34	0.55 (0.54, 0.56)	0.00	0.51 (0.49, 0.51)	0.00	4.58 (4.48, 4.67)	0.00	5.35 (5.23, 5.46)	0.00
25–29	0.59 (0.58, 0.6)	0.00	0.56 (0.55, 0.57)	0.00	3.22 (3.15, 3.28)	0.00	3.53 (3.46, 3.61)	0.00
20–24	0.70 (0.69, 0.71)	0.00	0.71 (0.69, 0.71)	0.00	1.93 (1.89, 1.97)	0.00	1.98 (1.93, 2.02)	0.00
15–19	Ref.							
Year								
2012–2016	0.81 (0.78, 0.84)	0.00	0.79 (0.76, 0.83)	0.00	4.72 (4.41, 5.05)	0.00	3.64 (3.37, 3.94)	0.00
2008–<2012	0.71 (0.68, 0.74)	0.00	0.72 (0.68, 0.76)	0.00	3.96 (3.69, 4.25)	0.00	2.85 (2.63, 3.09)	0.00
2004–<2008	1.05 (1.01, 1.09)	0.02	1.12 (1.05, 1.15)	0.00	3.48 (3.25, 3.73)	0.00	2.17 (2.01, 2.35)	0.00
2000–<2004	0.91 (0.86, 0.95)	0.00	0.96 (0.9, 1.01)	0.12	1.76 (1.63, 1.91)	0.00	1.33 (1.21, 1.45)	0.00
1996–<2000	Ref.							

1 Values are ORs (95% CIs).

### Projection

**FIGURE 2:**
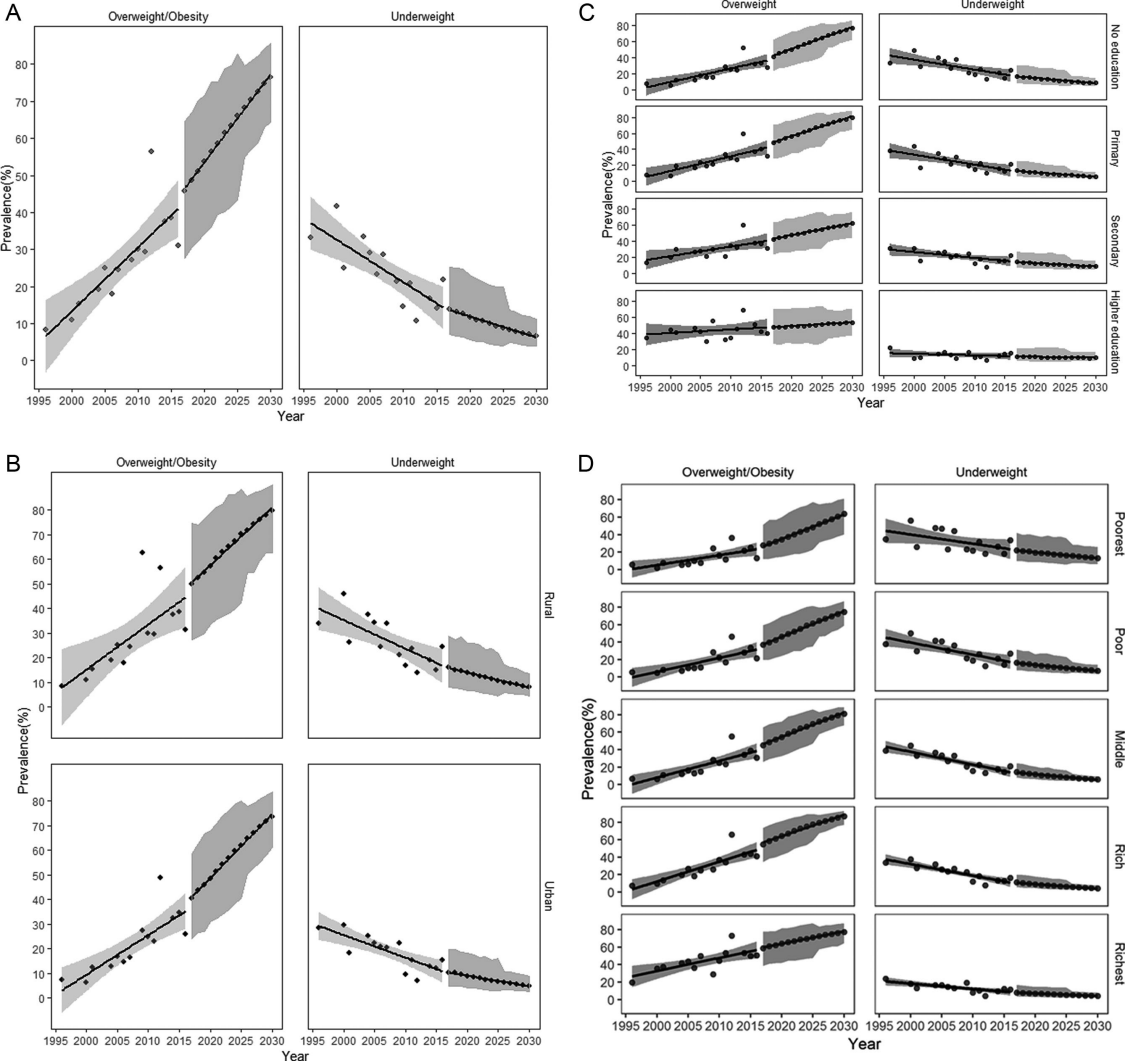
Projected weight trends in South and Southeast Asia. (A) Projection of underweight and overweight/obesity in South and Southeast Asia by 2030. (B) Place of residence–specific prevalence of underweight and overweight/obesity in South and Southeast Asia by 2030. (C) Education-specific prevalence of underweight and overweight/obesity in South and Southeast Asia by 2030. (D) Wealth index–specific prevalence of underweight and overweight/obesity in South and Southeast Asia by 2030.

If the current trend of underweight and overweight/obesity continues until 2030, the projected proportion of underweight and overweight/obesity in the region would be 6.6% (95% CI: 3.9%, 11.1%) and 76.6% (95% CI: 64.3%, 85.7%), respectively (Figure 2A). Projection of trends by place of residence suggest (Figure 2B) that by 2030 the prevalence of overweight/obesity in rural areas (79.8%; 95% CI: 62.5%, 90.2%) will exceed that of urban areas (73.6%; 95% CI: 61.1%,83.5%), as will the prevalence of underweight (rural = 7.9%; 95% CI: 4.5%, 13.4%; urban = 4.9%; 95% CI: 2.7%, 8.7%). Education-specific analysis estimated that by 2030 the prevalence of both underweight and overweight/obesity would be higher in those who had no education/primary education compared with those with a higher education (Figure 2C). Conversely, when the wealth index was used, specific projections suggested that by 2030 the prevalence of underweight would be higher among the poorest group and overweight/obesity higher among the richest group (Figure 2D).

Country-specific projections for Bangladesh and Nepal projected a 2030 prevalence of underweight of 5.7% (95% CI: 5.4%, 6.1%) in Bangladesh and 12.4% (95% CI: 10.4%, 14.4%) in Nepal, overweight/obesity projections were 83.5% (95% CI: 81.3.5%, 85.6%) and 60.3% (95% CI: 56.8%, 63.3%) for Bangladesh and Nepal, respectively. Projections stratified by determinants for these countries showed similar results to those for the overall sample, with higher projected prevalence of both underweight and overweight/obesity in rural areas and those with no education/primary education, along with a higher prevalence of overweight/obesity in the richest groups and underweight in the poorest groups **(Supplemental Figures 2 and 3)**.

## Discussion

This is the first comprehensive study evaluating trends (1996–2016) in the prevalence of underweight and overweight/obesity among the women in South and Southeast Asian countries, based on nationally representative surveys. This study found that among South and Southeast Asian women the mean BMI has increased 9% in the past 4 decades and the prevalence of overweight/obesity now exceeds the prevalence of underweight. Projection of these trends estimates that by 2030 two-thirds of the female population in this region will be overweight/obese and just under 10% underweight, with those in rural areas and those with no education at a greater future risk of both conditions. Country-specific analyses demonstrated large inequalities between countries in the prevalence of both overweight/obesity and underweight, although past and projected trends in those countries for which sufficient data points were available were similar.

Our findings for mean BMI agree with global trends in mean BMI and prevalence of overweight and obesity between 1975 and 2016 (23). They are also broadly consistent with those from previous studies that have demonstrated a significant decline in underweight in LMICs (24, 25). Our findings shows that place of residence, wealth index, education, and age group are strong determinants for both underweight and overweight/obesity also agree with findings from studies in both LMICs (26) and industrialized nations (27), with wealth found to be a major determinant of overweight/obesity in a number of studies (28, 29). Previous studies have also reported that most women gain weight during reproductive life, with this often linked to pregnancy, with many women unable to go back to their prepregnancy weight (30). However, pregnancy is not the only cause of weight gain in women of this age (31).

According to our findings for the period 1996–2014, the AARR of underweight was 1.3% and the AARI of overweight and obesity was 8.4%. In addition, according to country-specific findings, a larger AARR for underweight was found in urban areas, among wealthier families, and for those who had a higher education. A larger AARI for overweight was found in rural areas, among the poorest families, and for those who had a higher education.

Our findings suggest that by 2030 10% of the adult female population in South and Southeast Asia will be underweight, whereas more than two-thirds will be overweight or obese. We also found that a more rapid shift in both these conditions will be found in rural areas and among the poorest groups. Although our study only considered women in this region, this trend is likely to affect the rest of the population and across generations, as children of overweight mothers are at greater risk to becoming overweight themselves (9). This has grave ramifications for global health, with an increasing prevalence of high BMI likely to lead to increases in noncommunicable diseases (NCDs) (32, 33). These predicted dramatic increases in overweight and obesity and subsequent increases in NCDs will place heavy tolls on individuals, families, economies, and health care systems (12). It is for this reason that the prevention of NCDs is recognized as a central agenda in both health and development policy, including as a target in the SDGs (34). However, results from this study suggest that countries will struggle to meet global targets in obesity and NCDs if current trends continue.

The major strength of this study is the use of population-based nationally representative samples covering both rural and urban areas of 8 South and Southeast Asian countries, following the same sampling design and methodology in each country. BMI was based on measured height and weight and used Asian-specific cutoff points. However, this study suffers from some important limitations. Firstly, all data were cross-sectional in nature, which limits our ability to argue the casual directions of the associations we observed. Repeated cross-sectional data are useful for investigating population-level trends in prevalence, however, which was the main aim of the study. In addition, heterogeneity was found in both the years of available data between countries and in the sample characteristics. Another limitation for this study, which is common in data collection of this type, is that certain demographic information is self-reported (e.g., age and education status). Although this could introduce some bias, all height and weight measurements were collected by trained staff following standardized procedures, ensuring none were self-reported, thereby avoiding bias in the outcome measures. Changes in sample characteristics may reflect population changes in these countries, such as the increasing proportion of the sample living in urban areas over time. To counter this, some analyses were stratified by these characteristics and they were controlled for in regression analyses.

In conclusion, we found a gradual decline in underweight prevalence and a similar increase in overweight, among women in South and Southeast Asian countries, from 1996 to 2016. Our results also suggest that age, place of residence, education, and wealth are strong determinants of both these conditions. Modeled estimates forecast that by 2030 the prevalence of the DBM will be higher in rural areas and for those who have no education. It is imperative, in light of these findings, that countries implement effective and sustainable approaches to minimize the DBM. Failure to do so will result in countries failing to achieve time-bound health and development targets.

## Supplementary Material

nzz026_Supplement_files

## Notes

The authors reported no funding received for this study.

Author disclosures: TB, NT, RSM, MSI, MMH, and AM, no conflicts of interest.

Supplemental Tables 1–16 and Supplemental Figures 1–3 are available from the “Supplementary data” link in the online posting of the article and from the same link in the online table of contents at https://academic.oup.com/cdn/.

Abbreviations used: AARI, average annual rate of increase; AARR, average annual rate of reduction; DBM, double burden of malnutrition; DHS, Demographic and Health Survey; LMIC, low- and middle-income country; NCD, noncommunicable disease; SDG, Sustainable Development Goal.
