# Supplementary material for: Current Progress and Future Directions in the Double Burden of Malnutrition among Women in South and Southeast Asian Countries
Source: Curr Dev Nutr. 2019 May 16;3(7):nzz026. doi: 10.1093/cdn/nzz026 (PMC6584112; doi:10.1093/cdn/nzz026)
Supplement: nzz026_Supplement_files [file nzz026_supplement_files.zip › Supplimental figure.pdf]

Supplementary Data

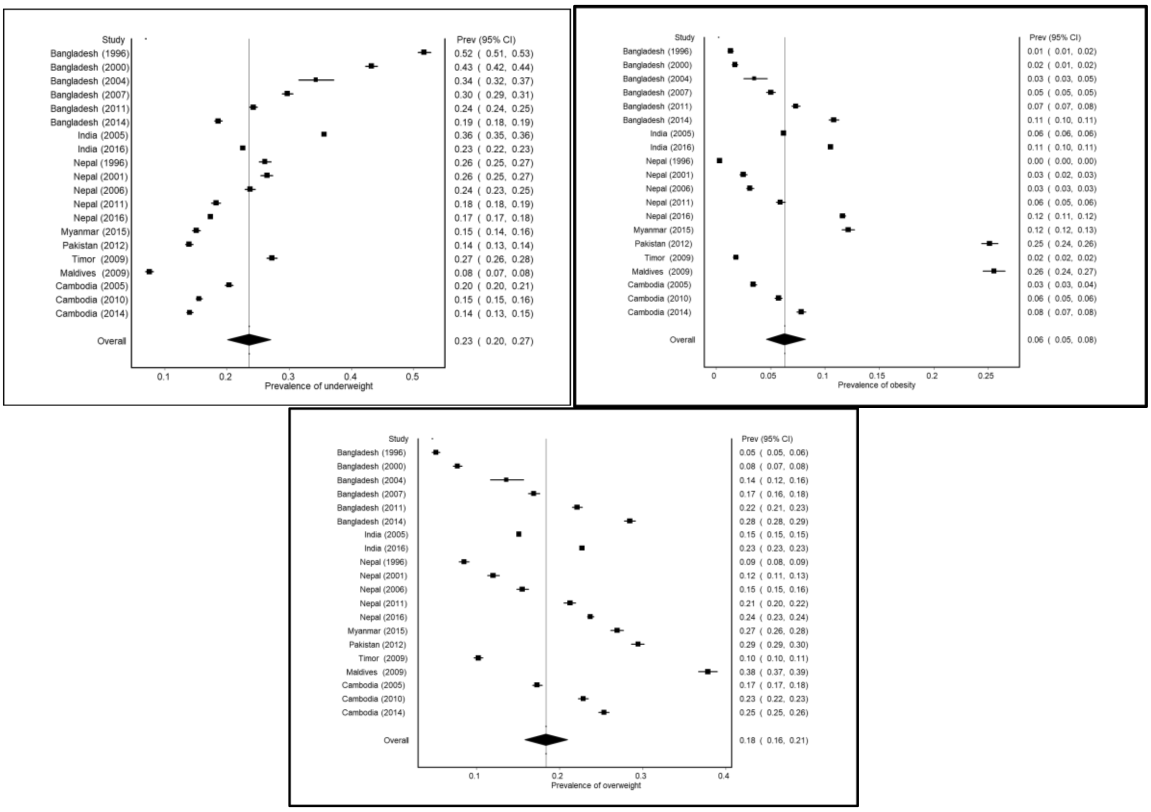

Supplementary figure 1: Trend of underweight, overweight and obesity on South and Southeast Asian country

Supplementary Data

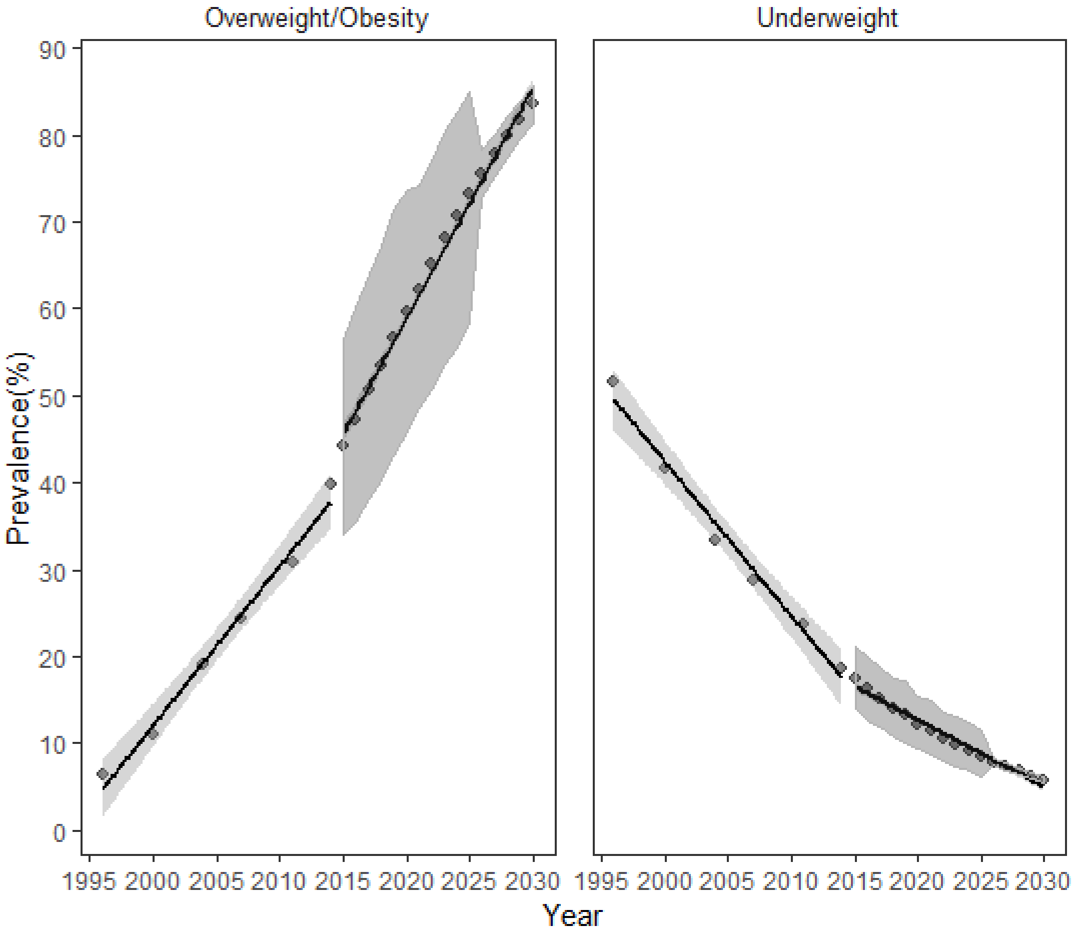

Supplementary figure 2: Projection of underweight and overweight in Bangladesh by 2030

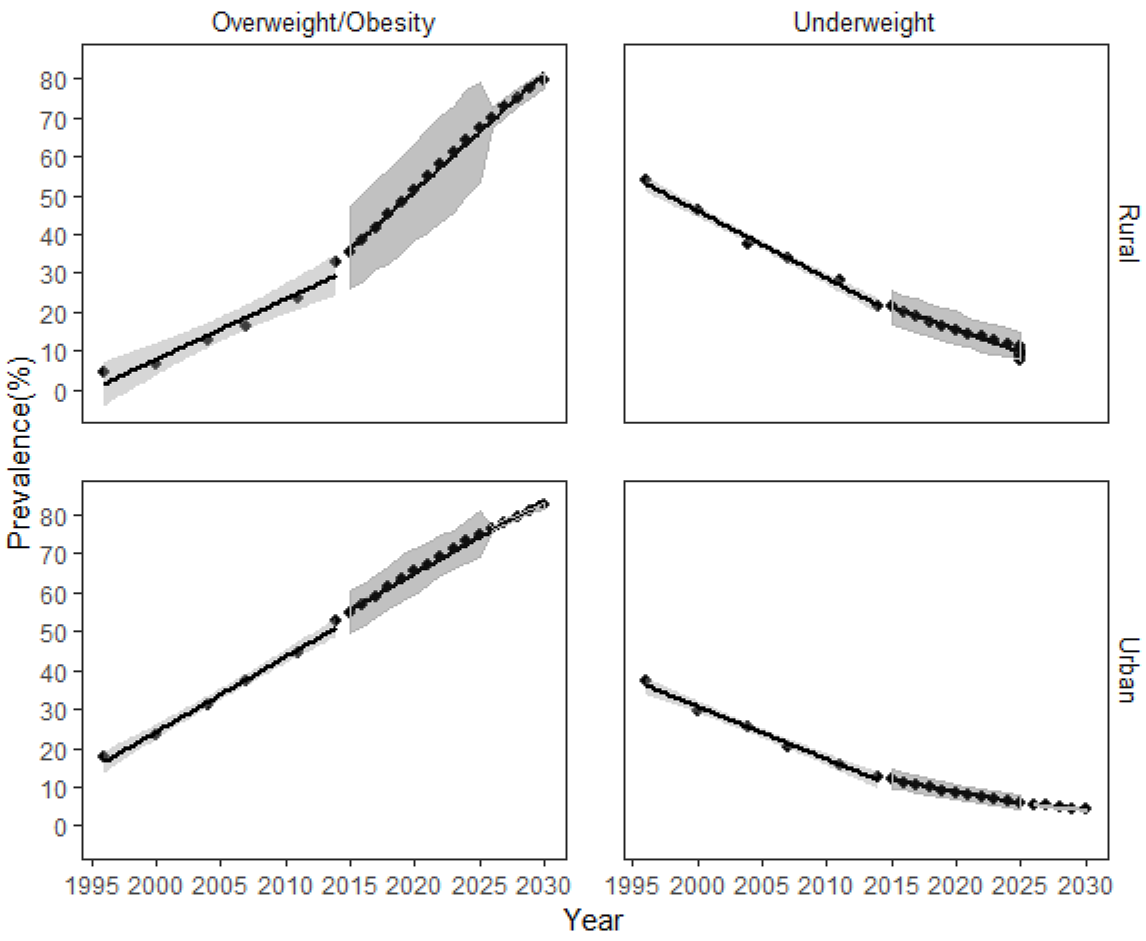

Supplementary figure 2.a: Place of residence specific prevalence of underweight and overweight in Bangladesh by 2030

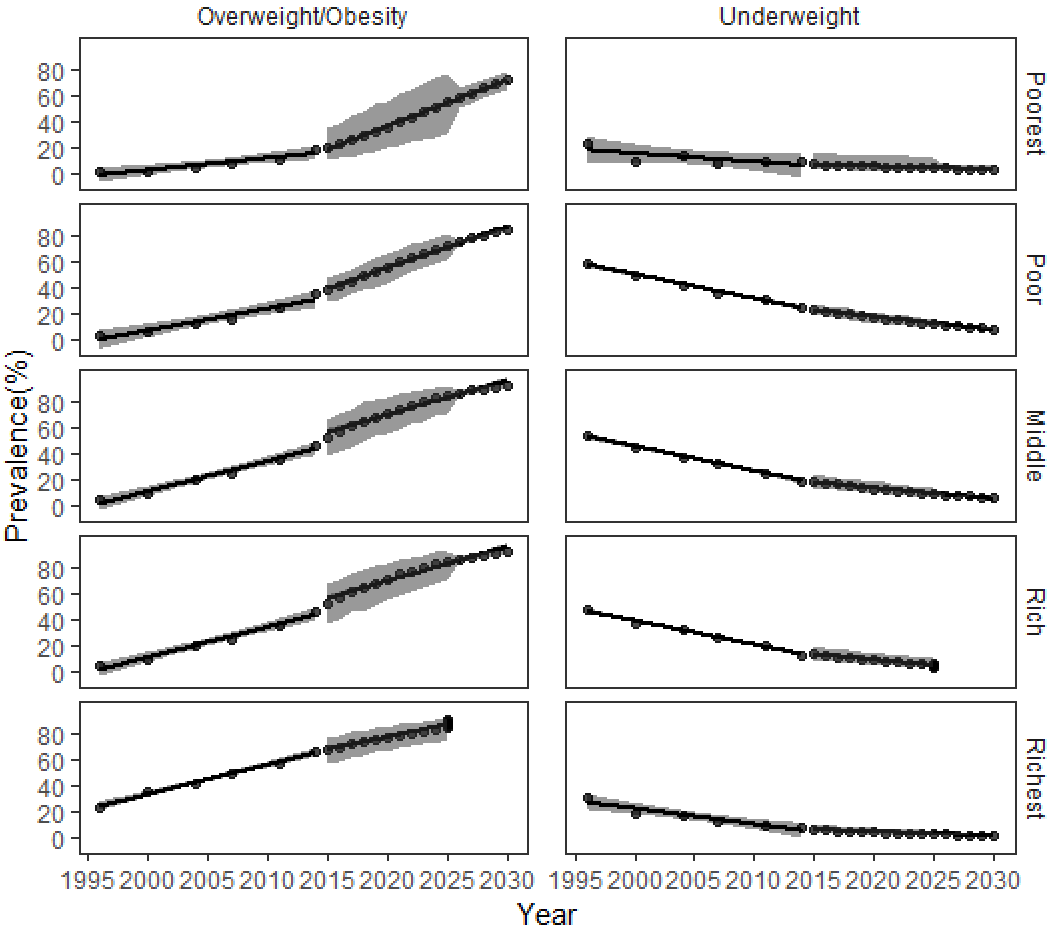

Supplementary figure 2.b: Education specific prevalence of underweight and overweight in Bangladesh by 2030

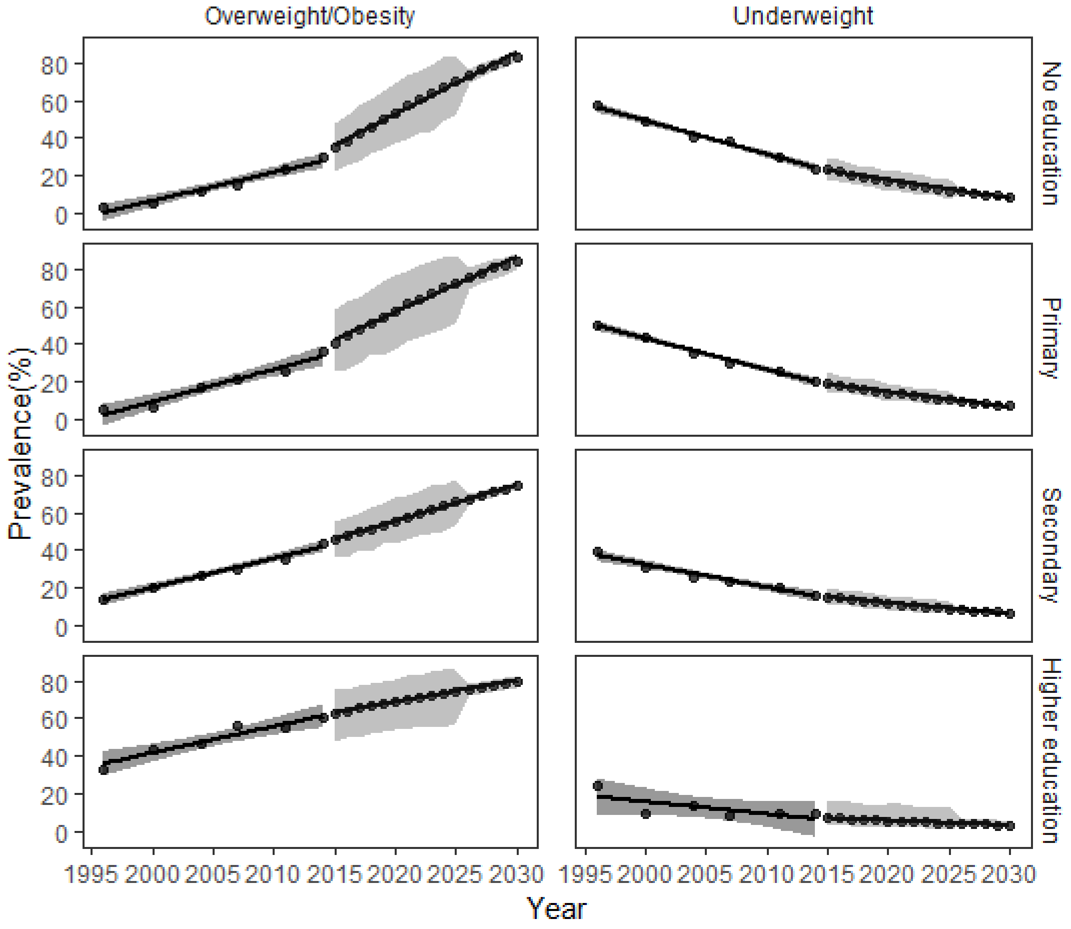

Supplementary figure 2.c: Education specific prevalence of underweight and overweight in Bangladesh by 2030

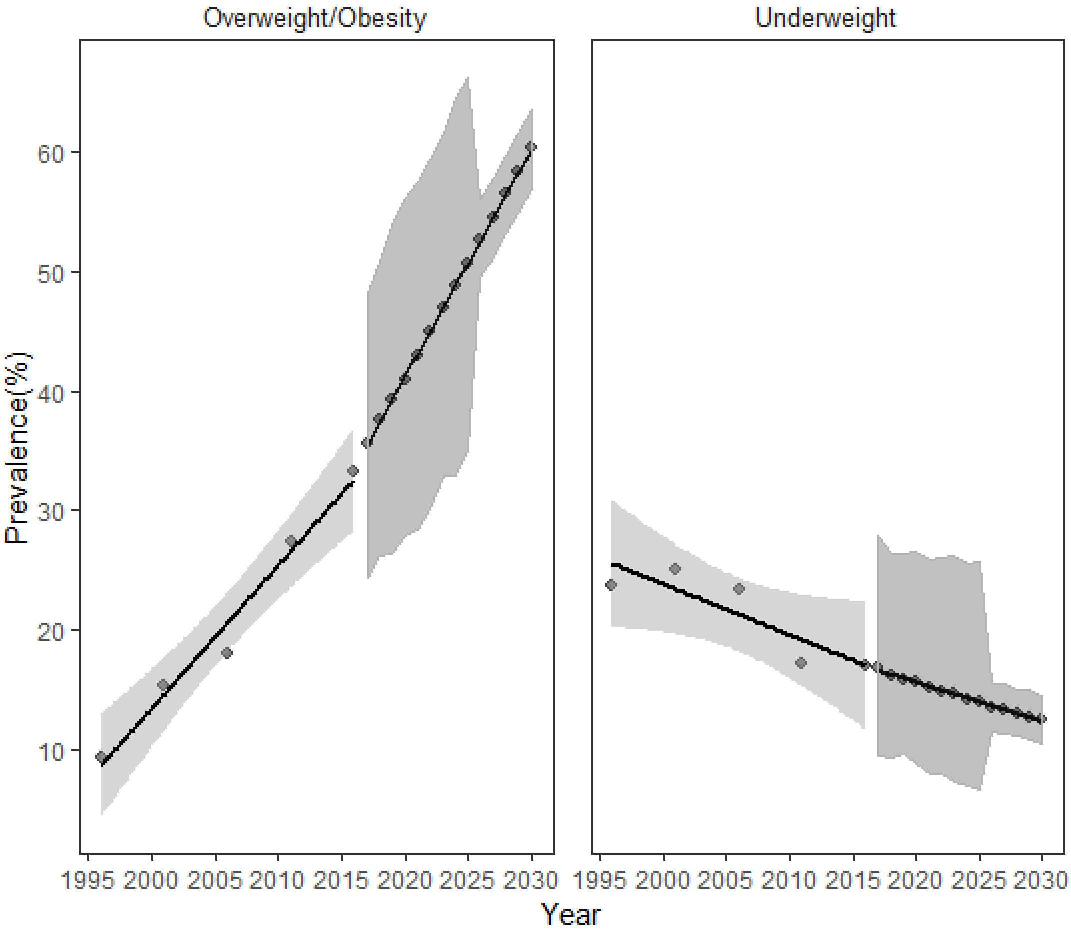

Supplementary figure 3: Projection of underweight and overweight in Nepal by 2030

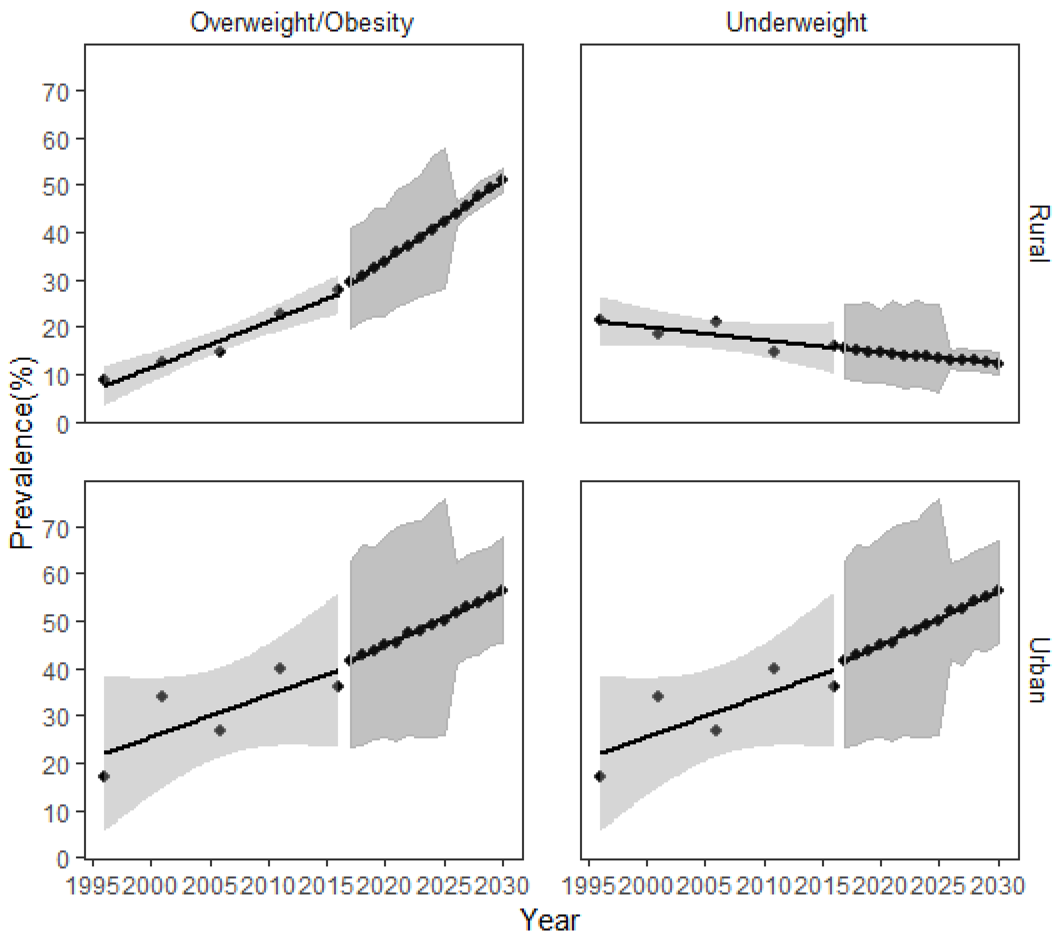

Supplementary figure 3.a: Place of residence specific prevalence of underweight and overweight in Nepal by 2030

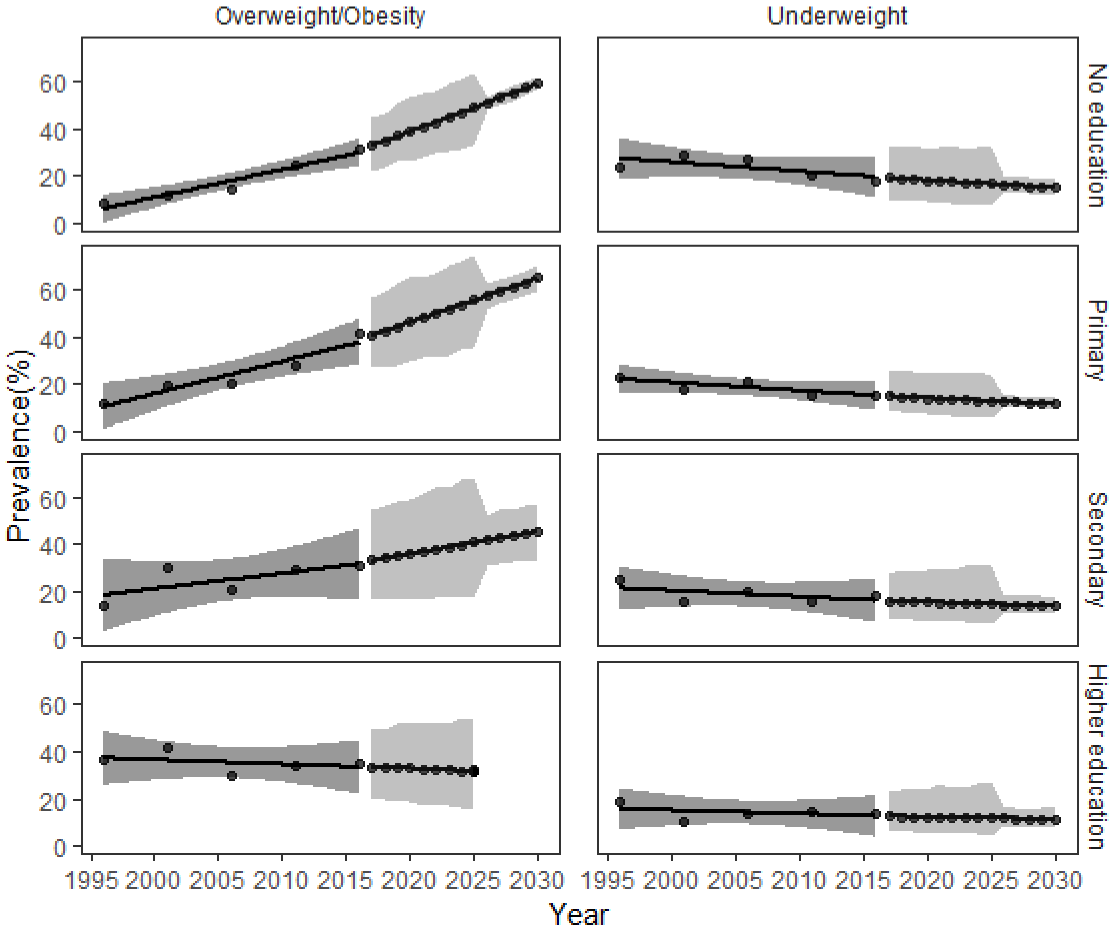

Supplementary figure 3.b: Wealth index specific prevalence of underweight and overweight in Nepal by 2030

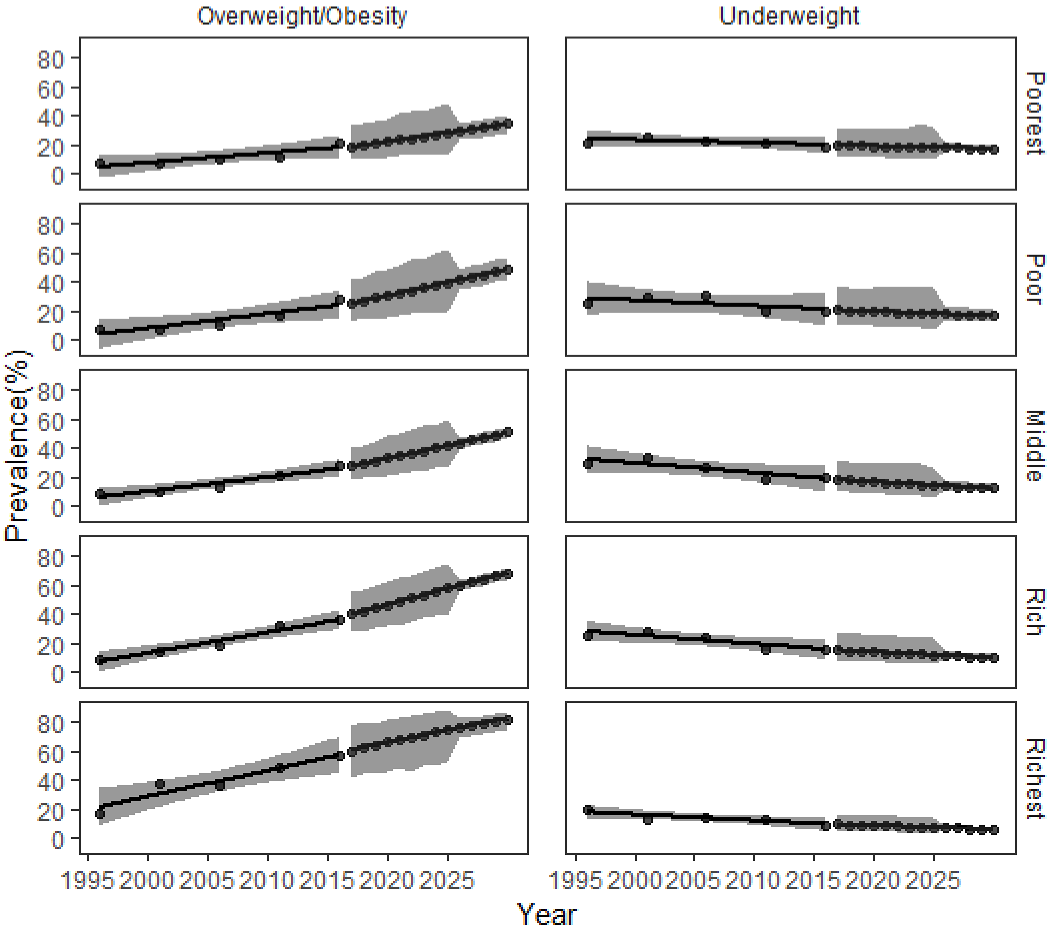

Supplementary figure 3.c: Education specific prevalence of underweight and overweight in Nepal by 2030
