# Supplementary material for: Current Progress and Future Directions in the Double Burden of Malnutrition among Women in South and Southeast Asian Countries
Source: Curr Dev Nutr. 2019 May 16;3(7):nzz026. doi: 10.1093/cdn/nzz026 (PMC6584112; doi:10.1093/cdn/nzz026)
Supplement: nzz026_Supplement_files [file nzz026_supplement_files.zip › Supplymentary tables.pdf]

Online Supporting Material

Supplementary Table-1.1: Average annual rate of reduction (AARR) in the prevalence of underweight among the Bangladeshi women 1996-2014

**Variables**	**Underweight**											
**Type of residence**	**1996**	**2000**	**AARR (1996-2000)**	**2004**	**AARR (2000-2004)**	**2007**	**AARR (2004-2007)**	**2011**	**AARR (2007-2011)**	**2014**	**AARR (2011-2014)**	**AARR (1996-2014)**
**National**	51.5	41.6	5.0	33.4	5.0	28.7	5.0	23.6	5.0	18.6	8.0	6.0
Urban	35.2	29.2	5.0	25	4.0	19.6	8.0	13.5	9.0	12.2	3.0	6.0
Rural	53.6	46.1	4.0	37.1	5.0	32.6	4.0	28	4.0	21.1	9.0	5.0
**Wealth index**												
Lowest quintile	64.6	56.1	3.0	47.1	4.0	43.4	3.0	40.1	2.0	32.2	7.0	4.0
Second quintile	58.3	51	3.0	40.5	6.0	35.4	4.0	30.2	4.0	24.9	6.0	5.0
Middle quintile	53.5	44.4	5.0	35.6	5.0	32.7	3.0	25.6	6.0	19	9.0	6.0
Fourth quintile	47.5	37.9	5.0	31.3	5.0	25.2	7.0	19.5	6.0	12.3	14.0	8.0
Highest quintile	31.6	19	12.0	17.2	2.0	13.4	8.0	8.4	11.0	7	6.0	8.0
**Education**												
No education	57.2	49.6	4.0	40.1	5.0	37.6	2.0	29.8	6.0	24.1	7.0	5.0
Primary	50.2	45.3	3.0	34.9		30.2	5.0	26.6	3.0	20.7	8.0	5.0
Secondary	39.2	32.9	4.0	27.2	5.0	23.6	5.0	20.6	3.0	15.2	10.0	5.0
Higher	28.1	11	21.0	16.2	-10.0	9.7	16.0	8.7	3.0	8.8	0.0	6.0
**Age**									0.0			
15-19	50.2	48.2	1.0	39.6	5.0	34.9	4.0	36.1	-1.0	31	5.0	3.0
20-24	53.2	41.5	6.0	36.1	3.0	31.7	4.0	28.2	3.0	22.7	7.0	5.0
25-29	48.7	38.6	6.0	30.9	5.0	28.5	3.0	21.5	7.0	16.8	8.0	6.0
30-34	53	41.2	6.0	31.3	7.0	24.6	8.0	20.1	5.0	13.5	12.0	8.0
35-39	58.4	51.9	3.0	30.1	13.0	27.3	3.0	19.7	8.0	13.5	12.0	8.0
40-44	49.7	55.3	-3.0	34.4	11.0	29.5	5.0	22	7.0	17.8	7.0	5.0
45-49	61.7	58	2.0	40.1	9.0	33.5	6.0	25.7	6.0	19.8	8.0	6.0

Supplementary Table-1.2. Average annual rate of increase (AARI) in the prevalence of overweight among the Bangladeshi women 1996-2014

Variables											
**Type of residence**	**1996**	**2000**	**AARI (1996-2000)**	**2004**	**AARI (2000-2004)**	**2007**	**AARI (2004-2007)**	**2011**	**AARI (2007-2011)**	**2014**	**AARR (2011-2014)**	**AARI (1996-2014)**
**National**	6.4	10.9	14.0	19.1	15.0	24.4	9.0	30.8	6.0	39.8	9.0	11.0
Urban	21.7	24.1	3.0	30.5	6.0	38.8	8.0	45.3	4.0	53.2	6.0	5.0
Rural	4.6	6.3	8.0	13.2	20.0	16.8	8.0	23.7	9.0	33.6	12.0	12.0
**Wealth index**												
Lowest quintile	1.9	1.7	-3.0	5.7	35.0	7.7	11.0	11.2	10.0	17.8	17.0	14.0
Second quintile	2.9	4.4	11.0	7.3	13.0	11.1	15.0	16.4	10.0	27.3	19.0	14.0
Middle quintile	3.8	6.2	13.0	12.8	20.0	14.8	5.0	24.5	13.0	34.7	12.0	13.0
Fourth quintile	4.5	9.6	21.0	20.3	21.0	24.9	7.0	35.4	9.0	47.2	10.0	14.0
Highest quintile	23.6	34.7	10.0	39.2	3.0	48.4	7.0	55.1	3.0	64.7	5.0	6.0
**Education**												
No education	3.7	5.1	8.0	11.5	23.0	14.1	7.0	22.4	12.0	30	10.0	12.0
Primary	5.4	5.9	2.0	15.9	28.0	20.4	9.0	25.4	6.0	35.2	11.0	11.0
Secondary	13.1	17.5	8.0	23.4	8.0	26.6	4.0	33.8	6.0	44.5	10.0	7.0
Higher	30	39.9	7.0	42.4	2.0	51.8	7.0	52.8	0.0	58.5	3.0	4.0
**Age**												
15-19	2.2	3.2	10.0	5.8	16.0	8	11.0	10.2	6.0	16.8	18.0	12.0
20-24	5.6	8.4	11.0	11.6	8.0	16	11.0	20.4	6.0	29.6	13.0	10.0
25-29	8.8	12.9	10.0	17.9	9.0	23.7	10.0	31.6	7.0	40	8.0	9.0
30-34	8.1	13.1	13.0	21	13.0	25	6.0	36.4	10.0	47.7	9.0	10.0
35-39	6.2	9.1	10.0	23.2	26.0	27.6	6.0	34.7	6.0	47.6	11.0	12.0
40-44	6.2	9.9	12.0	23.1	24.0	26.8	5.0	36.3	8.0	45.3	8.0	11.0
45-49	7.2	6.7	-2.0	21	33.0	25.8	7.0	32.3	6.0	41.7	9.0	11.0

Supplementary Table-1.3. Average annual rate of reduction (AARR) in the prevalence of underweight among the Indian women 2005-2016

Variables	2005	2016	Percent AARR (2005-2016)
**National**	29.6	21.9	3.0
**Type of residence**			
Urban	25	15.3	4.0
Rural	40.6	26.2	4.0
**Wealth index**			0.0
Lowest quintile	51.5	34.8	4.0
Second quintile	46.3	28.8	4.0
Middle quintile	38.3	22.6	5.0
Fourth quintile	28.9	16.8	5.0
Highest quintile	18.2	11.4	4.0
**Education**			
No education	41.7	24.3	5.0
Primary	34.9	21.7	4.0
Secondary	32.4	23.4	3.0
Higher	19.3	15.6	2.0
**Age**			
15-19	46.8	41.1	1.0
20-24	40.8	27.5	4.0
25-29	35.3	20.4	5.0
30-34	32.9	16.6	6.0
35-39	28.9	14.4	6.0
40-44	27.1	14	6.0
45-49	25.6	13.3	6.0

Supplementary Table-1.4. Average annual rate of increase (AARI) in the prevalence of overweight among the Indian women 2005-2016

Variables	2005	2016	Percent AARI (2005-2016)
**National**	25.5	31.1	2.0
**Type of residence**			
Urban	35.4	46.3	2.0
Rural	14.4	26.4	6.0
**Wealth index**			0.0
Lowest quintile	5.2	13.4	9.0
Second quintile	8.9	22	9.0
Middle quintile	15.3	31.8	7.0
Fourth quintile	26.4	42.9	5.0
Highest quintile	44.4	52.4	2.0
**Education**			
No education	13.9	28.6	7.0
Primary	21	34.2	5.0
Secondary	25.4	33.4	3.0
Higher	40.8	41.2	0.0
**Age**			
15-19	6.1	9.7	4.0
20-24	12.2	20.7	5.0
25-29	19.7	32.3	5.0
30-34	25.5	41.1	4.0
35-39	30.8	45.7	4.0
40-44	34.8	49.4	3.0
45-49	37.2	50.8	3.0

Supplementary Table-1.5. Average annual rate of reduction (AARR) in the prevalence of underweight among the Nepalese women 1996-2016

Variables	1996	2001	AARR (1996-2001)	2006	AARR (2001-2006)	2011	AARR (2006-2011)	2016	AARR (2011-2016)	AARR (1996-2016)
**National**	23.7	25.1	-1.0	23.4	1.0	17.2	6.0	17	0.0	2.0
**Type of residence**			0.0		0.0		0.0		0.0	0.0
Urban	21.2	16.7	5.0	16.1	1.0	14.1	3.0	15.7	-2.0	1.0
Rural	26.4	27.4	-1.0	25.1	2.0	18.8	6.0	20	-1.0	1.0
**Wealth index**										
Lowest quintile	23.8	27.2	-3.0	23.7	3.0	21.5	2.0	19.1	2.0	1.0
Second quintile	27.7	31.1	-2.0	32.1	-1.0	21.2	8.0	21.1	0.0	1.0
Middle quintile	31.9	34.5	-2.0	27.8	4.0	21.5	5.0	21.3	0.0	2.0
Fourth quintile	28.1	29.4	-1.0	23.3	5.0	16.6	7.0	17.3	-1.0	2.0
Highest quintile	20.3	13	9.0	12.4	1.0	11.9	1.0	8.6	6.0	4.0
**Education**										
No education	26.3	30.1	-3.0	28.2	1.0	22.6	4.0	18.6	4.0	2.0
Primary	24.8	17.8	6.0	20.9	-3.0	15.5	6.0	17.1	-2.0	2.0
Secondary	26	15.4	10.0	17.6	-3.0	15.5	3.0	18	-3.0	2.0
Higher	22.8	10.8	14.0	13.2	-4.0	13.8	-1.0	12.4	2.0	3.0
**Age**										
15-19	30.4	22.6	6.0	25.3	-2.0	25.8	0.0	30.3	-3.0	0.0
20-24	26.5	22.9	3.0	21	2.0	17.6	3.0	20	-3.0	1.0
25-29	23.3	22.6	1.0	23.1	0.0	20.8	2.0	15.1	6.0	2.0
30-34	24.9	25.9	-1.0	19.8	5.0	12.2	9.0	11	2.0	4.0
35-39	27.6	28.7	-1.0	24.2	3.0	12.3	13.0	10.4	3.0	5.0
40-44	27.5	30.5	-2.0	25.8	3.0	13.1	13.0	13	0.0	4.0
45-49	40	34.8	3.0	28.3	4.0	19.3	7.0	12.2	9.0	6.0

Supplementary Table-1.6. Average annual rate of increase (AARI) in the prevalence of overweight among the Nepalese women 1996-2016

Variables	1996	2001	AARI (1996-2001)	2006	AARI (2001-2006)	2011	AARI (2006-2011)	2016	AARI (2011-2016)	AARR (1996-2016)
National	9.3	15.3	10.0	17.9	3.0	27.4	9.0	33.2	4.0	6.6
**Type of residence**										
Urban	17.5	36.7	16.0	34.1	-1.0	42.3	4.0	39.7	-1.0	4.4
Rural	8.2	12.1	8.0	15.7	5.0	24.6	9.0	27.9	3.0	6.3
**Wealth index**			0.0		0.0		0.0		0.0	0.0
Lowest quintile	7.2	7.7	1.0	10.7	7.0	12.1	2.0	23.5	14.0	6.2
Second quintile	7.1	8.1	3.0	10.8	6.0	16.3	9.0	28.4	12.0	7.2
Middle quintile	7.9	10.8	6.0	12.6	3.0	21.3	11.0	27	5.0	6.4
Fourth quintile	8.2	13.1	10.0	18.2	7.0	29.1	10.0	36.2	4.0	7.7
Highest quintile	16.5	36.5	17.0	38.3	1.0	49.8	5.0	58	3.0	6.7
**Education**			0.0		0.0		0.0		0.0	
No education	7.7	11.1	8.0	15.3	7.0	23.7	9.0	32.8	7.0	7.5
Primary	11.4	18.1	10.0	21.1	3.0	28.2	6.0	42.4	8.0	6.8
Secondary	12.6	28.8	18.0	21.9	-5.0	28.8	6.0	32.9	3.0	5.2
Higher	34.8	40.1	3.0	28	-7.0	34.2	4.0	39.5	3.0	0.7
**Age**			0.0		0.0		0.0		0.0	
15-19	6.2	8.4	6.0	10.5	5.0	10.4	0.0	12.4	4.0	3.6
20-24	9.5	10.2	1.0	15.2	8.0	18.5	4.0	23.1	5.0	4.6
25-29	9.3	13.4	8.0	20.2	9.0	28.1	7.0	38.4	6.0	7.4
30-34	9	18.1	15.0	22.5	4.0	38.6	11.0	46	4.0	8.6
35-39	9.3	15.7	11.0	24	9.0	38.6	10.0	49.3	5.0	8.7
40-44	7	19.5	23.0	24.9	5.0	39.6	10.0	49.1	4.0	10.5
45-49	4.7	16.5	29.0	23.1	7.0	35.4	9.0	49.1	7.0	12.8

Supplementary Table-1.7. Average annual rate of reduction (AARR) in the prevalence of underweight among the Cambodian women 2005-2014

Variables	2005	2010	AARR (2005-2010)	2014	AARR (2010-2014)	AARR (2005-2014)
**National**	19.9	14.7	6.0	13.9	1.0	4.0
**Type of residence**			0.0		0.0	0.0
Urban	17.3	9.6	11.0	13.5	-9.0	1.0
Rural	21	16.8	4.0	14.1	4.0	4.0
**Wealth index**			0.0		0.0	0.0
Lowest quintile	23.9	21.7	2.0	15.3	8.0	5.0
Second quintile	22.5	18.7	4.0	14.6	6.0	5.0
Middle quintile	22.8	15.2	8.0	14.3	2.0	5.0
Fourth quintile	17.6	12.7	6.0	13.5	-2.0	2.0
Highest quintile	16.6	9.2	11.0	12.7	-8.0	1.0
**Education**			0.0		0.0	0.0
No education	19.1	17.8	1.0	10.9	12.0	6.0
Primary	20.4	15.4	5.0	12.4	5.0	5.0
Secondary	21	13.8	8.0	16.7	-5.0	2.0
Higher	23.9	13.1	11.0	18.8	-9.0	1.0
**Age**			0.0		0.0	0.0
15-19	28	25.1	2.0	27.5	-2.0	0.0
20-24	19.2	22.8	-3.0	20.2	3.0	0.0
25-29	18.7	16.6	2.0	13.9	4.0	3.0
30-34	16.7	12.8	5.0	7.4	13.0	9.0
35-39	18.4	13.3	6.0	6.8	15.0	11.0
40-44	16.2	13.9	3.0	7.7	14.0	8.0
45-49	20.6	14.9	6.0	9.2	11.0	9.0

Supplementary Table-1.8. Average annual rate of increase (AARI) in the prevalence of underweight among the Cambodian women 2005-2014

Variables	2005	2010	Percent AARI (2005-2014)	2014	Percent AARI (2010-2014)	Percent AARI (1996-2014)
**National**	20.1	30	8.0	33.8	3.49	1.79
**Type of residence**			0.0			
Urban	27.3	40.5	8.0	36.5	3.59	1.84
Rural	19.2	25.7	6.0	32.3	3.82	1.94
**Wealth index**			0.0			
Lowest quintile	11.5	16.4	7.0	26.1	3.34	1.71
Second quintile	13.9	24.6	12.0	31.1	3.00	1.56
Middle quintile	15.6	24.3	9.0	32.3	3.32	1.71
Fourth quintile	24.4	34.4	7.0	35.3	3.72	1.90
Highest quintile	32.7	42.1	5.0	38.8	4.23	2.14
**Education**			0.0			
No education	20	25.8	5.0	38.9	4.22	2.14
Primary	21.5	28.3	6.0	36.9	4.06	2.06
Secondary	19.6	30.9	10.0	27.3	3.11	1.60
Higher	15.2	34.3	18.0	22.8	2.37	1.27
**Age**			0.0			
15-19	7.7	12.6	10.0	10.3	2.16	1.13
20-24	13.6	8.9	-8.0	19.4		-0.04
25-29	16.4	17.6	1.0	28.6	5.70	2.86
30-34	24	27.9	3.0	39.3	4.99	2.51
35-39	31.2	35.6	3.0	49.5	5.56	2.79
40-44	29.8	39.4	6.0	48.7	4.40	2.23
45-49	33	38.2	3.0	50.7	5.43	2.73

Supplementary Table-2.1: Multinominal logistic regression analysis of odds ratio of being underweight and being overweight compare with normal BMI category for Bangladesh

Variables	Unadjusted				Adjusted			
	Underweight		Overweight/obesity		Underweight		Overweight/obesity	
Type of residence	OR 95% CI	p value	OR 95% CI	p value	OR 95% CI	p value	OR 95% CI	p value
Urban	0.68 (0.66-0.72)	0.00	2.41 (2.32-2.51)	0.00	0.92 (0.87-0.96)	0.00	1.35 (1.29-1.42)	0.00
Rural	Ref							
**Wealth index**								
Highest quintile	0.37 (0.34-0.39)	0.00	6.67 (6.2-7.18)	0.00	0.43 (0.4-0.47)	0.00	4.75 (4.36-5.18)	0.00
Fourth quintile	0.56 (0.53-0.59)	0.00	3.13 (2.9-3.38)	0.00	0.62 (0.58-0.66)	0.00	2.57 (2.37-2.79)	0.00
Middle quintile	0.66 (0.63-0.7)	0.00	2 (1.85-2.17)	0.00	0.7 (0.66-0.75)	0.00	1.78 (1.64-1.94)	0.00
Second quintile	0.77 (0.73-0.82)	0.00	1.37 (1.26-1.49)	0.00	0.79 (0.74-0.83)	0.00	1.31 (1.2-1.43)	0.00
Lowest quintile	Ref							
**Education**								
Higher	0.33 (0.3-0.37)	0.00	4.15 (3.85-4.46)	0.00	0.58 (0.51-0.65)	0.00	2.08 (1.9-2.27)	0.00
Secondary	0.59 (0.56-0.62)	0.00	2.12 (2.02-2.23)	0.00	0.77 (0.73-0.82)	0.00	1.75 (1.65-1.87)	0.00
Primary	0.79 (0.75-0.82)	0.00	1.36 (1.29-1.43)	0.00	0.88 (0.84-0.93)	0.00	1.28 (1.21-1.36)	0.00
No education	Ref							
**Age**								
45-49	0.93 (0.86-1.01)	0.10	4.9 (4.41-5.43)	0.00	0.92 (0.84-1.01)	0.08	5.52 (4.92-6.18)	0.00
40-44	0.82 (0.76-0.89)	0.00	5.21 (4.72-5.77)	0.00	0.79 (0.73-0.86)	0.00	5.94 (5.33-6.63)	0.00
35-39	0.76 (0.71-0.82)	0.00	4.83 (4.38-5.33)	0.00	0.71 (0.66-0.78)	0.00	5.51 (4.96-6.13)	0.00
30-34	0.75 (0.7-0.8)	0.00	4.46 (4.05-4.91)	0.00	0.68 (0.63-0.73)	0.00	4.96 (4.48-5.5)	0.00
25-29	0.78 (0.73-0.84)	0.00	3.52 (3.2-3.87)	0.00	0.71 (0.66-0.76)	0.00	3.75 (3.39-4.14)	0.00
20-24	0.9 (0.84-0.96)	0.00	2.17 (1.97-2.39)	0.00	0.87 (0.81-0.93)	0.00	2.14 (1.93-2.37)	0.00
15-19	Ref							
**Year**								
2014	0.37 (0.34-0.4)	0.00	6.31 (5.5-7.23)	0.00	0.36 (0.33-0.4)	0.00	5.28 (4.52-6.17)	0.00
2011	0.42 (0.39-0.46)	0.00	4.46 (3.89-5.11)	0.00	0.43 (0.39-0.47)	0.00	3.42 (2.92-3.99)	0.00
2007	0.5 (0.46-0.54)	0.00	3.43 (2.98-3.95)	0.00	0.52 (0.47-0.57)	0.00	2.44 (2.08-2.86)	0.00
2004	0.57 (0.53-0.62)	0.00	2.64 (2.29-3.05)	0.00	0.58 (0.54-0.64)	0.00	1.99 (1.69-2.33)	0.00
2000	0.72 (0.66-0.78)	0.00	1.51 (1.29-1.78)	0.00	0.72 (0.65-0.79)	0.00	1.41 (1.17-1.69)	0.00
1996	Ref							

Supplementary Table-2.2: Multinominal logistic regression analysis of odds ratio of being underweight and being overweight compare with normal BMI category for India

Variables	Unadjusted				Adjusted			
	Underweight		Overweight/obesity		Underweight		Overweight/obesity	
	OR 95% CI	p value	OR 95% CI	p value	OR 95% CI	p value	OR 95% CI	p value
**Type of residence**								
Urban	0.79 (0.78-0.8)	0.00	2.03 (2.01-2.06)	0.00	0.98 (0.96-0.99)	0.00	1.29 (1.27-1.31)	0.00
Rural	Ref							
**Wealth index**								
Highest quintile	0.49 (0.48-0.5)	0.00	5.43 (5.32-5.53)	0.00	0.49 (0.48-0.5)	0.00	4.65 (4.54-4.76)	0.00
Fourth quintile	0.62 (0.61-0.63)	0.00	3.76 (3.69-3.84)	0.00	0.62 (0.61-0.63)	0.00	3.42 (3.35-3.5)	0.00
Middle quintile	0.71 (0.69-0.72)	0.00	2.53 (2.48-2.58)	0.00	0.7 (0.69-0.72)	0.00	2.43 (2.38-2.48)	0.00
Second quintile	0.83 (0.81-0.84)	0.00	1.67 (1.64-1.7)	0.00	0.83 (0.82-0.85)	0.00	1.64 (1.61-1.68)	0.00
Lowest quintile	Ref							
**Education**								
Higher	0.64 (0.62-0.65)	0.00	1.69 (1.66-1.72)	0.00	0.77 (0.75-0.79)	0.00	1.25 (1.23-1.28)	0.00
Secondary	0.93 (0.92-0.95)	0.00	1.25 (1.23-1.26)	0.00	0.83 (0.81-0.84)	0.00	1.37 (1.35-1.39)	0.00
Primary	0.9 (0.89-0.92)	0.00	1.21 (1.19-1.23)	0.00	0.86 (0.85-0.88)	0.00	1.19 (1.17-1.22)	0.00
No education	Ref							
**Age**								
45-49	0.49 (0.48-0.5)	0.00	6.91 (6.75-7.08)	0.00	0.43 (0.42-0.44)	0.00	8.52 (8.3-8.74)	0.00
40-44	0.5 (0.49-0.52)	0.00	6.53 (6.38-6.69)	0.00	0.44 (0.43-0.45)	0.00	7.92 (7.72-8.12)	0.00
35-39	0.49 (0.48-0.5)	0.00	5.62 (5.5-5.75)	0.00	0.43 (0.42-0.44)	0.00	6.71 (6.55-6.87)	0.00
30-34	0.52 (0.51-0.53)	0.00	4.71 (4.6-4.81)	0.00	0.47 (0.46-0.48)	0.00	5.36 (5.24-5.49)	0.00
25-29	0.56 (0.55-0.57)	0.00	3.28 (3.21-3.36)	0.00	0.53 (0.52-0.54)	0.00	3.5 (3.42-3.58)	0.00
20-24	0.67 (0.66-0.68)	0.00	1.94 (1.89-1.98)	0.00	0.68 (0.67-0.69)	0.00	1.94 (1.89-1.99)	0.00
15-19	Ref							
**Year**								
2016	0.71 (0.7-0.72)	0.00	1.17 (1.15-1.19)	0.00	0.64 (0.63-0.65)	0.00	1.55 (1.52-1.57)	0.00
2005	Ref							

Supplementary Table-2.3: Multinominal logistic regression analysis of odds ratio of being underweight and being overweight compare with normal BMI category for Nepal

Variables	Unadjusted				Adjusted			
	Underweight		Overweight/obesity		Underweight		Overweight/obesity	
**Type of residence**	**OR 95% CI**	**p value**	**OR 95% CI**	**p value**	**OR 95% CI**	**p value**	**OR 95% CI**	**p value**
Urban	0.95 (0.89-1.01)	0.08	2.62 (2.48-2.78)	0.00	1.11 (1.03-1.2)	0.01	1.15 (1.07-1.24)	0.00
Rural	**Ref**							
**Wealth index**								
Highest quintile	0.85 (0.78-0.94)	0.00	5.13 (4.69-5.62)	0.00	0.93 (0.84-1.04)	0.22	4.38 (3.92-4.88)	0.00
Fourth quintile	1.14 (1.05-1.24)	0.00	2.27 (2.06-2.5)	0.00	1.2 (1.1-1.31)	0.00	2.07 (1.87-2.3)	0.00
Middle quintile	1.28 (1.18-1.39)	0.00	1.61 (1.45-1.78)	0.00	1.31 (1.21-1.43)	0.00	1.47 (1.32-1.63)	0.00
Second quintile	1.26 (1.16-1.37)	0.00	1.35 (1.21-1.49)	0.00	1.28 (1.18-1.39)	0.00	1.27 (1.14-1.41)	0.00
Lowest quintile	**Ref**							
**Education**								
Higher	0.61 (0.53-0.7)	0.00	2.41 (2.17-2.68)	0.00	0.67 (0.57-0.78)	0.00	1.31 (1.15-1.5)	0.00
Secondary	0.76 (0.71-0.81)	0.00	1.78 (1.67-1.9)	0.00	0.72 (0.66-0.78)	0.00	1.64 (1.5-1.79)	0.00
Primary	0.76 (0.7-0.82)	0.00	1.6 (1.48-1.72)	0.00	0.75 (0.69-0.82)	0.00	1.63 (1.49-1.78)	0.00
No education	**Ref**							
**Age**								
45-49	1.24 (1.11-1.38)	0.00	3.6 (3.18-4.06)	0.00	0.92 (0.81-1.04)	0.19	5.92 (5.13-6.83)	0.00
40-44	1.08 (0.97-1.2)	0.15	3.63 (3.23-4.06)	0.00	0.81 (0.72-0.91)	0.00	5.97 (5.22-6.83)	0.00
35-39	0.95 (0.87-1.05)	0.36	3.26 (2.92-3.64)	0.00	0.74 (0.66-0.83)	0.00	5.2 (4.57-5.9)	0.00
30-34	0.83 (0.76-0.91)	0.00	2.78 (2.49-3.09)	0.00	0.67 (0.61-0.75)	0.00	4.09 (3.62-4.62)	0.00
25-29	0.82 (0.75-0.89)	0.00	2.05 (1.84-2.28)	0.00	0.68 (0.62-0.75)	0.00	2.93 (2.6-3.3)	0.00
20-24	0.81 (0.74-0.88)	0.00	1.42 (1.27-1.58)	0.00	0.73 (0.66-0.8)	0.00	1.76 (1.56-1.99)	0.00
15-19	**Ref**							
**Year**								
2016	0.96 (0.87-1.07)	0.47	4.78 (4.22-5.42)	0.00	0.94 (0.82-1.06)	0.30	3.79 (3.25-4.43)	0.00
2011	0.88 (0.79-0.98)	0.02	3.55 (3.13-4.03)	0.00	0.88 (0.78-0.99)	0.03	2.65 (2.28-3.07)	0.00
2006	1.12 (1.03-1.23)	0.01	2.19 (1.94-2.47)	0.00	1.08 (0.98-1.2)	0.14	1.73 (1.5-1.99)	0.00

2001	1.19 (1.08-1.3)	0.00	1.84 (1.62-2.09)	0.00	1.17 (1.06-1.3)	0.00	1.28 (1.1-1.48)	0.00
1996	**Ref**							

Supplementary Table-2.4 Multinominal logistic regression analysis of odds ratio of being underweight and being overweight compare with normal BMI category for Pakistan

**Variables**	**Unadjusted**				**Adjusted**			
	**Underweight**		**Overweight/obesity**		**Underweight**		**Overweight/obesity**	
**Type of residence**	**OR 95% CI**	**p value**	**OR 95% CI**	**p value**	**OR 95% CI**	**p value**	**OR 95% CI**	**p value**
Urban	0.69 (0.55-0.88)	0.00	1.85 (1.61-2.12)	0.00	0.81 (0.62-1.07)	0.14	1.1 (0.93-1.31)	0.26
Rural	**Ref**							
**Wealth index**								
Highest quintile	0.45 (0.3-0.68)	0.00	4.01 (3.2-5.01)	0.00	0.58 (0.35-0.97)	0.04	3.49 (2.59-4.69)	0.00
Fourth quintile	0.73 (0.52-1.03)	0.07	3.1 (2.47-3.89)	0.00	0.85 (0.57-1.27)	0.43	2.96 (2.27-3.86)	0.00
Middle quintile	1.06 (0.78-1.44)	0.72	2.17 (1.73-2.73)	0.00	1.15 (0.82-1.6)	0.41	2.16 (1.69-2.76)	0.00
Second quintile	0.75 (0.55-1.02)	0.07	1.4 (1.12-1.75)	0.00	0.79 (0.58-1.08)	0.15	1.36 (1.08-1.71)	0.01
Lowest quintile	**Ref**							
**Education**								
Higher	0.66 (0.43-1.01)	0.06	1.87 (1.49-2.35)	0.00	0.88 (0.53-1.44)	0.61	1.06 (0.8-1.4)	0.68
Secondary	0.74 (0.53-1.02)	0.07	1.33 (1.1-1.61)	0.00	0.85 (0.58-1.23)	0.38	0.95 (0.75-1.19)	0.63
Primary	0.93 (0.67-1.28)	0.64	1.32 (1.08-1.62)	0.01	0.95 (0.68-1.34)	0.78	1.07 (0.86-1.33)	0.56
No education	**Ref**							
**Age**								
45-49	1.13 (0.65-1.95)	0.67	7.36 (4.78-11.35)	0.00	1.15 (0.66-2.02)	0.63	6.61 (4.23-10.32)	0.00
40-44	1.17 (0.68-2.02)	0.57	7 (4.54-10.78)	0.00	1.18 (0.68-2.06)	0.55	6.3 (4.04-9.83)	0.00
35-39	0.83 (0.48-1.44)	0.51	6.54 (4.28-9.99)	0.00	0.85 (0.49-1.48)	0.57	6 (3.88-9.26)	0.00
30-34	1.27 (0.76-2.13)	0.37	5.98 (3.91-9.14)	0.00	1.3 (0.77-2.19)	0.33	5.44 (3.52-8.39)	0.00
25-29	1.52 (0.92-2.49)	0.10	3.71 (2.43-5.65)	0.00	1.59 (0.96-2.62)	0.07	3.2 (2.08-4.92)	0.00
20-24	1.63 (0.99-2.68)	0.06	2.51 (1.63-3.88)	0.00	1.64 (1-2.71)	0.05	2.36 (1.52-3.67)	0.00
15-19	**Ref**							

Supplementary Table-2.5 Multinominal logistic regression analysis of odds ratio of being underweight and being overweight compare with normal BMI category for Myanmar

**Variables**	**Unadjusted**				**Adjusted**			
	**Underweight**		**Overweight/obesity**		**Underweight**		**Overweight/obesity**	
**Type of residence**	**OR 95% CI**	**p value**	**OR 95% CI**	**p value**	**OR 95% CI**	**p value**	**OR 95% CI**	**p value**
Urban	1.04 (0.92-1.17)	0.57	1.76 (1.62-1.91)	0.00	0.97 (0.84-1.13)	0.70	1.37 (1.23-1.52)	0.00
Rural	**Ref**							
**Wealth index**								
Highest quintile	0.99 (0.84-1.17)	0.88	2.98 (2.62-3.39)	0.00	0.84 (0.68-1.04)	0.11	2.55 (2.17-3.01)	0.00
Fourth quintile	0.91 (0.77-1.07)	0.23	2.3 (2.02-2.61)	0.00	0.8 (0.67-0.96)	0.02	2.12 (1.84-2.44)	0.00
Middle quintile	0.97 (0.82-1.13)	0.67	1.9 (1.67-2.16)	0.00	0.89 (0.75-1.05)	0.15	1.82 (1.59-2.09)	0.00
Second quintile	0.83 (0.7-0.97)	0.02	1.45 (1.27-1.65)	0.00	0.79 (0.67-0.93)	0.01	1.4 (1.22-1.6)	0.00
Lowest quintile	**Ref**							
**Education**								
Higher	1.22 (0.98-1.52)	0.08	1.5 (1.27-1.76)	0.00	1.31 (1.01-1.69)	0.04	0.94 (0.78-1.14)	0.53
Secondary	1.25 (1.06-1.48)	0.01	1.23 (1.09-1.4)	0.00	1.18 (0.97-1.43)	0.09	1.33 (1.15-1.54)	0.00
Primary	0.96 (0.81-1.14)	0.63	1.33 (1.18-1.51)	0.00	0.98 (0.82-1.17)	0.80	1.25 (1.1-1.43)	0.00
No education	**Ref**							
**Age**								
45-49	0.79 (0.65-0.97)	0.02	6.5 (5.46-7.73)	0.00	0.86 (0.7-1.05)	0.14	7.18 (5.98-8.61)	0.00
40-44	0.54 (0.44-0.67)	0.00	6.13 (5.17-7.26)	0.00	0.57 (0.46-0.71)	0.00	6.94 (5.81-8.3)	0.00
35-39	0.53 (0.44-0.65)	0.00	5.7 (4.82-6.74)	0.00	0.56 (0.45-0.69)	0.00	6.55 (5.5-7.81)	0.00
30-34	0.6 (0.5-0.72)	0.00	4.38 (3.7-5.17)	0.00	0.62 (0.51-0.75)	0.00	5.04 (4.24-5.99)	0.00
25-29	0.66 (0.55-0.79)	0.00	3.07 (2.59-3.64)	0.00	0.67 (0.56-0.8)	0.00	3.46 (2.9-4.12)	0.00
20-24	0.89 (0.76-1.04)	0.14	1.81 (1.51-2.16)	0.00	0.89 (0.76-1.05)	0.17	1.93 (1.61-2.32)	0.00
15-19	**Ref**							

Supplementary Table-2.6. Multinominal logistic regression analysis of odds ratio of being underweight and being overweight compare with normal BMI category for Maldives

Variables	Unadjusted				Adjusted			
	Underweight		Overweight/obesity		Underweight		Overweight/obesity	
**Type of residence**	**OR 95% CI**	**p value**	**OR 95% CI**	**p value**	**OR 95% CI**	**p value**	**OR 95% CI**	**p value**
Urban	0.76 (0.53-1.09)	0.14	1.24 (1.04-1.49)	0.02	1.01 (0.53-1.94)	0.97	1.44 (1.05-1.97)	0.02
Rural	**Ref**							
**Wealth index**								
Highest quintile	0.62 (0.39-0.99)	0.05	1.19 (0.93-1.51)	0.17	0.6 (0.27-1.31)	0.20	0.91 (0.61-1.35)	0.63
Fourth quintile	0.64 (0.44-0.93)	0.02	1.19 (0.98-1.46)	0.08	0.55 (0.36-0.84)	0.01	1.2 (0.96-1.51)	0.12
Middle quintile	0.72 (0.53-0.97)	0.03	1.05 (0.88-1.25)	0.60	0.65 (0.48-0.89)	0.01	1.13 (0.94-1.35)	0.19
Second quintile	0.91 (0.67-1.22)	0.52	1.17 (0.98-1.4)	0.08	0.86 (0.63-1.17)	0.34	1.25 (1.04-1.49)	0.02
Lowest quintile	**Ref**							
**Education**								
Higher	1.73 (0.86-3.46)	0.12	0.58 (0.39-0.86)	0.01	1.2 (0.53-2.71)	0.66	0.86 (0.55-1.35)	0.52
Secondary	2.24 (1.64-3.08)	0.00	0.47 (0.4-0.55)	0.00	1.14 (0.69-1.87)	0.61	0.84 (0.66-1.08)	0.17
Primary	1.39 (0.99-1.95)	0.06	0.81 (0.69-0.94)	0.01	0.99 (0.64-1.53)	0.95	1.13 (0.93-1.37)	0.23
No education	**Ref**							
**Age**								
45-49	0.3 (0.14-0.65)	0.00	4.79 (2.74-8.39)	0.00	0.34 (0.14-0.82)	0.02	4.11 (2.26-7.47)	0.00
40-44	0.26 (0.13-0.54)	0.00	3.87 (2.24-6.7)	0.00	0.29 (0.13-0.66)	0.00	3.3 (1.85-5.9)	0.00
35-39	0.25 (0.13-0.5)	0.00	3.03 (1.76-5.21)	0.00	0.28 (0.13-0.59)	0.00	2.53 (1.44-4.45)	0.00
30-34	0.32 (0.16-0.63)	0.00	2.76 (1.6-4.74)	0.00	0.36 (0.18-0.73)	0.00	2.38 (1.37-4.14)	0.00
25-29	0.49 (0.26-0.93)	0.03	1.87 (1.09-3.2)	0.02	0.53 (0.28-1.01)	0.05	1.72 (1-2.97)	0.05
20-24	0.88 (0.47-1.64)	0.68	1.41 (0.82-2.43)	0.21	0.91 (0.49-1.69)	0.76	1.37 (0.8-2.37)	0.25
15-19	**Ref**							

Supplementary Table-2.7: Multinominal logistic regression analysis of odds ratio of being underweight and being overweight compare with normal BMI category for Timor

Variables	Unadjusted				Adjusted			
	Underweight		Overweight/obesity		Underweight		Overweight/obesity	
**Type of residence**	**OR 95% CI**	**p value**	**OR 95% CI**	**p value**	**OR 95% CI**	**p value**	**OR 95% CI**	**p value**
Urban	1.05 (0.95-1.16)	0.34	1.82 (1.61-2.06)	0.00	1.14 (1.02-1.27)	0.02	1.23 (1.06-1.42)	0.01
Rural	**Ref**							
**Wealth index**								
Highest quintile	0.84 (0.73-0.96)	0.01	3.72 (3.04-4.54)	0.00	0.84 (0.71-0.98)	0.03	3.26 (2.59-4.11)	0.00
Fourth quintile	1.04 (0.92-1.18)	0.53	2.2 (1.79-2.71)	0.00	1.05 (0.92-1.2)	0.51	2.14 (1.73-2.66)	0.00
Middle quintile	0.96 (0.84-1.09)	0.50	1.4 (1.12-1.74)	0.00	0.96 (0.84-1.09)	0.54	1.4 (1.12-1.76)	0.00
Second quintile	0.99 (0.87-1.13)	0.86	1.24 (0.99-1.57)	0.06	0.99 (0.87-1.13)	0.86	1.26 (1-1.59)	0.05
Lowest quintile	**Ref**							
**Education**								
Higher	0.68 (0.49-0.95)	0.02	1.9 (1.35-2.69)	0.00	0.65 (0.46-0.92)	0.01	1.11 (0.76-1.62)	0.58
Secondary	1 (0.9-1.1)	0.93	1.24 (1.07-1.43)	0.00	0.87 (0.77-0.98)	0.02	1.38 (1.15-1.64)	0.00
Primary	1.02 (0.91-1.14)	0.76	1.39 (1.18-1.63)	0.00	0.96 (0.85-1.08)	0.49	1.52 (1.28-1.8)	0.00
No education	**Ref**							
**Age**								
45-49	0.78 (0.66-0.91)	0.00	3.4 (2.68-4.32)	0.00	0.72 (0.6-0.85)	0.00	4.39 (3.38-5.69)	0.00
40-44	0.84 (0.72-0.98)	0.03	3.9 (3.11-4.9)	0.00	0.79 (0.67-0.92)	0.00	4.65 (3.65-5.92)	0.00
35-39	0.7 (0.6-0.8)	0.00	3.52 (2.83-4.38)	0.00	0.67 (0.57-0.77)	0.00	3.99 (3.18-5.01)	0.00
30-34	0.75 (0.64-0.87)	0.00	3.54 (2.82-4.45)	0.00	0.73 (0.62-0.85)	0.00	3.72 (2.94-4.71)	0.00
25-29	0.72 (0.62-0.83)	0.00	2.76 (2.2-3.47)	0.00	0.7 (0.61-0.81)	0.00	2.89 (2.29-3.64)	0.00
20-24	0.98 (0.87-1.11)	0.78	1.76 (1.39-2.23)	0.00	0.99 (0.87-1.12)	0.82	1.79 (1.41-2.27)	0.00
15-19	**Ref**							

Supplementary Table-2.8: Multinominal logistic regression analysis of odds ratio of being underweight and being overweight compare with normal BMI category for Cambodia

Variables	Unadjusted				Adjusted			
	Underweight		Overweight/obesity		Underweight		Overweight/obesity	
**Type of residence**	**OR 95% CI**	**p value**	**OR 95% CI**	**p value**	**OR 95% CI**	**p value**	**OR 95% CI**	**p value**
Urban	0.83 (0.77-0.9)	0.00	1.59 (1.49-1.69)	0.00	0.92 (0.83-1.02)	0.12	1.05 (0.97-1.14)	0.24
Rural	Ref							
**Wealth index**								
Highest quintile	0.73 (0.66-0.82)	0.00	2.71 (2.47-2.98)	0.00	0.67 (0.59-0.77)	0.00	3.37 (2.99-3.8)	0.00
Fourth quintile	0.82 (0.73-0.92)	0.00	2.08 (1.88-2.31)	0.00	0.77 (0.69-0.87)	0.00	2.33 (2.09-2.6)	0.00
Middle quintile	0.91 (0.82-1.02)	0.11	1.43 (1.28-1.59)	0.00	0.88 (0.78-0.98)	0.02	1.54 (1.38-1.73)	0.00
Second quintile	0.92 (0.82-1.02)	0.12	1.3 (1.16-1.44)	0.00	0.9 (0.8-1.01)	0.06	1.35 (1.2-1.51)	0.00
Lowest quintile	Ref							
**Education**								
Higher	1.12 (0.91-1.38)	0.29	0.94 (0.78-1.13)	0.51	1.61 (1.27-2.04)	0.00	0.58 (0.47-0.71)	0.00
Secondary	1.03 (0.93-1.15)	0.57	1.05 (0.96-1.14)	0.32	1.12 (0.99-1.27)	0.07	0.88 (0.79-0.98)	0.02
Primary	0.98 (0.89-1.08)	0.65	1.15 (1.06-1.25)	0.00	1 (0.91-1.11)	0.98	1.09 (1-1.19)	0.06
No education	Ref							
**Age**								
45-49	0.74 (0.65-0.85)	0.00	6.92 (6.02-7.95)	0.00	0.75 (0.65-0.86)	0.00	7.61 (6.57-8.82)	0.00
40-44	0.62 (0.54-0.71)	0.00	6.25 (5.45-7.17)	0.00	0.62 (0.54-0.71)	0.00	7.04 (6.09-8.13)	0.00
35-39	0.57 (0.5-0.66)	0.00	5.92 (5.15-6.8)	0.00	0.57 (0.49-0.65)	0.00	6.76 (5.85-7.81)	0.00
30-34	0.47 (0.42-0.54)	0.00	4.32 (3.77-4.96)	0.00	0.48 (0.42-0.55)	0.00	4.41 (3.83-5.08)	0.00
25-29	0.59 (0.52-0.66)	0.00	2.66 (2.31-3.06)	0.00	0.59 (0.52-0.67)	0.00	2.74 (2.37-3.16)	0.00
20-24	0.71 (0.64-0.8)	0.00	1.72 (1.48-1.99)	0.00	0.71 (0.63-0.79)	0.00	1.78 (1.53-2.07)	0.00
15-19	Ref							
**Year**								
2014	0.8 (0.74-0.87)	0.00	1.93 (1.8-2.07)	0.00	0.79 (0.73-0.86)	0.00	2.04 (1.89-2.21)	0.00
2010	0.8 (0.73-0.88)	0.00	1.62 (1.49-1.76)	0.00	0.91 (0.82-1.01)	0.07	1.28 (1.17-1.39)	0.00
2005	Ref							
